# Investigation of colistin-resistant non-human *Acinetobacter seifertii* isolates

**DOI:** 10.1128/spectrum.02311-26

**Published:** 2026-09-17

**Authors:** Ellen M. E. Sykes, Yen Chi Dang, Dawn White, Valeria Mateo-Estrada, Anna Muzaleva, George Zhanel, Jeremy Dettman, Julie Chapados, Suzanne Gerdis, Izhar U. H. Khan, Santiago Castillo-Ramírez, Ayush Kumar

**Affiliations:** 1 Department of Microbiology, University of Manitoba 468335, Winnipeg, Canada; 2 Programa de Genómica Evolutiva, Centro de Ciencias Génomicas, Universidad Nacional Autónoma de México, Cuernavaca, Mexico; 3 Department of Medical Microbiology and Infectious Diseases, University of Manitoba 574854, Winnipeg, Canada; 4 Ottawa Research and Development Centre, Agriculture and Agri-Food Canada 98674, Ottawa, Canada; University of Minnesota Twin Cities, St. Paul, Minnesota, USA

**Keywords:** colistin resistance, lipidomics, *Acinetobacter calcoaceticus–baumannii* complex, emerging pathogens

## Abstract

The *Acinetobacter calcoaceticus–baumannii* (ACB) complex comprises multiple nosocomial infection-causing *Acinetobacter* species, the best-known being *Acinetobacter baumannii*. Once mainly considered opportunistic, *Acinetobacter* species are now being isolated from agricultural and food sources. We obtained and sequenced four *Acinetobacter* isolates from tank milk in Bogor, Indonesia. Using Average Nucleotide Identity and digital DNA:DNA hybridization, they were identified as *Acinetobacter seifertii*, a member of the ACB complex. Interestingly, all four isolates were resistant to colistin, a membrane-interacting antimicrobial polycationic peptide considered a drug of last resort. No known mechanism of colistin resistance was identified through analysis with the Comprehensive Antibiotic Resistance Database, and no known genetic substitutions that confer colistin resistance were found. Lipidomic analysis revealed lyso-phosphatidylethanolamine and an acyl-homoserine lactone quorum sensing molecule in significant abundance compared to colistin-sensitive *A. baumannii* ATCC 17978-VU. A membrane permeabilization assay with the four isolates showed muted interactions with colistin, and virulence-associated phenotypes did not vary greatly from the reference strain. Finally, the *A. seifertii* isolates were equally or less virulent than an *A. seifertii* reference strain in an insect infection model. Together, this suggests a potential unidentified mechanism of colistin resistance in *A. seifertii* or the discovery of tolerant isolates. Exploring non-human antibiotic-resistant bacterial species helps fill the gap in our understanding of the emergence of new pathogens.

**IMPORTANCE:**

Investigating drug-resistant bacterial species found outside of hospital settings is critical to understand and combat antibiotic resistance. Microbiological connections between humans, animals, and the environment can inform on how to use antimicrobial compounds appropriately, which in turn may curb the rise of bacterial antimicrobial resistance and failure of more antibiotics. *Acinetobacter* species are a group of soil- and water-dwelling microbes that are increasingly the cause of serious opportunistic infections, the best-known species being the multidrug-resistant pathogen *A. baumannii*. In this study, we discovered *A. seifertii* in tank milk in Indonesia that was resistant to a last-resort antibiotic, but the known genetic and cellular mechanisms of this resistance were not detected. Study and surveillance of such bacterial isolates will help us uncover and better understand bacterial antibiotic avoidance mechanisms and how pathogens emerge and move between different niches.

## INTRODUCTION

Members of the *Acinetobacter* genus have only recently been investigated for their clinical relevance. The term *Acinetobacter calcoaceticus–Acinetobacter baumannii* (ACB) complex coined in 1991 (1) currently includes six species with validly published names: *A. baumannii*, *A. pittii*, *A. nosocomialis*, *A. calcoaceticus*, and, most recently, *A. lactucae* and *A. seifertii* (2). The ACB member most widely reported to be associated with hospital-acquired infections is *A. baumannii*. Indeed, the World Health Organization has classified carbapenem-resistant *A. baumannii* as a critical priority pathogen for which more research and development is required (3). Study of this pathogen and its relatives is critical because upwards of 70% of *A. baumannii* strains are multidrug resistant, with some even resistant to last-resort antibiotics (4).

Characterization of *A. baumannii* and other ACB complex members has mainly been clinically focused, but there is mounting evidence that investigation into non-human strains (animal, environmental) is also essential (5). For example, emerging antibiotic-resistant *Acinetobacter* species *A. junii* (6) and *A. berenziniae* (7) are being considered as potential human pathogens. By exploring the diversity of non-human *Acinetobacter* species*,* further insight can be gathered regarding their evolution, including the acquisition and transfer of antibiotic resistance genes (8), as well as their potential to cause serious consequences for human health (9). *Acinetobacter seifertii* was first classified as a separate species within the ACB complex in 2015 (10). Compared to other *Acinetobacter* species, very little is understood about the population structure, global distribution, environmental niche, antibiotic resistance profile, and virulence potential of *A. seifertii*. More recently, though, it has been isolated from clinical settings in Japan (11), Brazil (12), and Taiwan (13). Interestingly, some clinical (11, 14, 15) and non-clinical (15) isolates have shown resistance to the last-resort antibiotic colistin.

Colistin is a glycopeptide antibiotic that was primarily used to treat nosocomial bacterial infections. Due to toxicity, its use has declined in human medicine but use in livestock and veterinary practices as a strategy for growth promotion and infection prevention among animals remains high mainly due to decreased efficacy of other antibiotics (16–18). Colistin resistance has been characterized best in clinical and reference strains of *A. baumannii* and is associated with the modulation of membrane homeostasis (19). This includes the modification or loss of outer membrane lipooligosaccharide (LOS), including the target of colistin, lipid A. Modification of lipid A is a common colistin resistance mechanism observed in many bacterial species (20, 21). In *A. baumannii,* the addition of phosphoethanolamine masks the negative charge of lipid A, thereby interfering with any potential interaction with cationic colistin and thus preventing membrane disruption (19). This resistance mechanism is governed by the two-component system PmrAB along with the phosphoethanolamine transferase PmrC (or the homolog EptA) (22, 23). Mobile colistin resistance (*mcr*) genes—of which *mcr-1* (24)*, mcr-2*, *mcr-3* (25), and *mcr-4* (26) have been detected in *A. baumannii—*are also known to add phosphoethanolamine to lipid A. Another resistance mechanism is the complete loss of LOS, which can be facilitated by insertion elements and mutations in membrane-associated biosynthetic pathways (27–30).

The mechanisms of colistin resistance in non-*baumannii* species of *Acinetobacter* have not been studied in detail but, due to phylogenetic relatedness, it is likely that they are common in species, such as *A. seifertii*. Here, we explore colistin resistance in non-clinical isolates of this ACB complex member with the investigation of four colistin-resistant *A. seifertii* isolates found in tank milk in Indonesia using lipidomics and phenotypic assays.

## MATERIALS AND METHODS

### Bacterial isolation and culture conditions

The *A. seifertii* isolates investigated in this study (with names shortened to IK22, IK23, IK24, and IK25 for simplicity) were part of an *Acinetobacter* species collection, of which many are *A. baumannii* from Sykes et al. (8). They were isolated from tank milk sources from Bogor, Indonesia. The milk samples were taken from separate tanks, and the cows were not receiving colistin treatment, though it is unknown if the cows were exposed to other compounds. Samples were first grown on tryptic soy agar with 5% sheep’s blood, followed by CHROMAgar *Acinetobacter* selective growth media (CHROMAgar, Micronostyx, Ottawa, Canada) each at 30°C (a permissible growth temperature) for 48 h. *A. seifertii* DSM 102854^T^ was purchased through the DSMZ culture library; *A. baumannii* ATCC 17978-VU was part of our lab culture collection. Liquid cultures needed for analyses of the new isolates, and reference strains were incubated in lysogeny broth (LB) (BD, New Jersey, USA) at 37°C (the optimal growth temperature for *Acinetobacter* species) for 18 h with shaking.

### Whole-genome sequencing

Genomic DNA was extracted from each *A. seifertii* isolate using the DNAeasy UltraClean Microbial Kit (Qiagen, MD, USA) from a culture inoculated with a pure colony of each isolate according to the manufacturer’s instructions. Sequence libraries were prepared and pooled using the DNA Prep Kit and the NextSeq 500 Mid Output Kit (Illumina, CA, USA). The Illumina NextSeq 500 platform at the Agriculture and Agri-Food Canada Ottawa Research and Development Center was used for whole-genome sequencing. SPAdes v 3.12.0 was used for *de novo* genome assembly (31). Completeness and quality of the genome assemblies were performed using QUAST v 5.0.2 (32) and CheckM v 1.0.11 (33) with 95% completeness and ≤5% contamination thresholds, respectively.

### Species determination

Average Nucleotide Identity by BLAST (ANIb) was used to determine the species of the isolates. All isolate assemblies (IK22, IK23, IK24, IK25) and reference genomes (*A. seifertii* UBA3035 [GCA_002367535.1], *A. seifertii* DSM 102854^T^ [GCF_000368065.1], *A. baumannii* ATCC 17978-VU [NZ_CP018664.1], and *A. baumannii* AB030 [SAMN26199121]) were uploaded to the Jspecies server (34). The digital DNA:DNA hybridization (dDDH) calculation was performed using The Type (Strain) Genome Server (35) using the same assemblies. Multi-locus sequence typing (MLST) was performed using the Pasteur scheme as reference (36) via ABRicate (37). When no MLST hits were observed, the genomes and novel sequence type (ST) profiles were uploaded to pubMLST.org for ST assignment. Phyloviz (38) was used to facilitate the goeBURST algorithm of analysis (39) to determine the Clonal Complex, an assignment based on STs that share identical alleles at six of seven loci.

### Minimum inhibitory concentration (MIC) determination

Antibiotic susceptibility was evaluated using the broth microdilution method as per the Clinical and Laboratory Standards Institute (CLSI) guidelines (40) and included three biological replicates. The full CANWARD panel (41) of antibiotics was used for testing. Based on CLSI breakpoints (40, 42), isolates were categorized into susceptible, intermediate, and resistant.

### Colistin resistome evaluation

Each isolate genome and reference strain (*A. baumannii* ATCC 17978-VU, *A. seifertii* DSM 102854^T^) sequence was analyzed using the Resistance Gene Identifier (RGI) in the Comprehensive Antibiotic Resistance Database (CARD) (43) using perfect, strict, and loose parameters. Percent identity was used to generate the heatmap in GraphPad Prism v 10.0.0. Additionally, mutations previously associated with colistin resistance in *A. baumannii* were investigated in all *A. seifertii* isolates through gene comparison using National Center for Biotechnology Information (NCBI) TBLASTN (44). Alignments were performed using MAFFT (45) and visualized using ESPript (46). Insertion sequences were evaluated using ISFinder (47) with an e-value cutoff of >0.01.

### Lipid extraction for mass spectrometry

Lipid extraction was carried out using a previously published protocol (48) with a few modifications. In brief, 250 mL of LB was inoculated and grown to OD_600_ of 0.8–1.0 with each *A. seifertii* isolate as well as *A. baumannii* ATCC 17978-VU and *A. seifertii* DSM 102854^T^. Cells were harvested and washed with 1x PBS. A single-layer Bligh–Dyer reaction was then achieved by adding chloroform, methanol, and water (1:2:0.8 v/v). After 20 min of incubation and centrifugation, an additional wash with the single-phase Bligh–Dyer mix was performed. Hydrolysis of LOS was performed with a mild acid buffer (50 mM sodium acetate pH 4.5, 1% SDS) and sonicated at a constant duty cycle for 20 s at 50% output. The samples were then boiled, and the solution was converted to a two-phase Bligh–Dyer reaction by adding chloroform, methanol, and water (2:2:1.8 v/v). The lower phase was extracted, and the upper phase was washed twice with a two-phase Bligh–Dyer solution. After pooling of the lower phases, the samples were rotary evaporated with a Buchi R-100 (Buchi, Newcastle, USA) controlled with an ARCTIC A25 Refrigerated Circulator Cooling System (Thermo Fisher Scientific). The samples were stored at −20°C prior to transport on dry ice to BioZone (University of Toronto, Toronto, Canada) for liquid chromatography–mass spectrometry analysis.

### Liquid chromatography–mass spectrometry analysis

The lipid extraction samples prepared above were resuspended in chloroform, transferred to a glass vial, and then dried under nitrogen. A 10-μL aliquot of each sample was run in a randomized order on a ZIC-polymeric hydrophilic interaction chromatography (pHILIC) column or a ZIC-hydrophilic interaction chromatography (HILIC) column (SeQuant, Millipore Sigma, Oakville, Canada) coupled to an Orbitrap or an Orbitrap Q Exactive mass spectrometer (Thermo Fisher Scientific) according to previously published methods (49). In brief, both positive and negative modes were used with a full scan range m/z 80–1,200, resolution 70,000 at 1 Hz, automatic gain control (AGC) target of 3 × 106, and a maximum injection time of 100 ms. Fragmentation of pHILIC column-separated metabolites was performed in a data-dependent manner on the Q Exactive (Thermo Fisher Scientific) mass spectrometer, with the five most intense ions picked in a 1 m/z exclusion window and at a normalized collision energy of 30. Raw spectral files were uploaded to MassiVE (https://massive.ucsd.edu/ProteoSAFe/static/massive.jsp?redirect=auth) (50). Statistical analysis was conducted in MetaboAnalyst v 5.0 (51). Principal component analysis was used to determine the variance in biological replicates (*n* = 3) for each sample using a 95% confidence interval (CI) for each cluster. Metabolite identification was performed using LIPIDMAPS (52) as the reference database via ChemSpider (https://www.chemspider.com/). In MetaboAnalyst, a one-way analysis of variance (ANOVA) with Fisher’s *post-hoc* testing was used to determine significant differences in lipids compared to *A. baumannii* ATCC 17978-VU. Enrichment analysis was performed using the Kyoto Encyclopedia of Genes and Genomes (KEGG; https://www.genome.jp/kegg/pathway.html) pathway categories.

### Membrane permeabilization assay

Membrane integrity in the presence of colistin (0–16 μg/mL) was assessed using the fluorescent, non-polar dye 1-N-phenylnaphthylamine (NPN) according to Sabnis et al. (53). In general, overnight cultures of the *A. seifertii* isolates and two reference strains (*A. baumannii* ATCC 17978-VU and *A. seifertii* DSM 102854^T^) were sub-cultured in fresh LB and grown until an OD_600_ of 0.4–0.5. One milliliter aliquots of the cultures were pelleted then washed with and resuspended in 5 mM HEPES buffer (pH 7.2) supplemented with 5 mM glucose. Aliquots (200 μL) were dispensed into black, optically clear, flat bottom 96-well plates. Then, NPN was added to a final concentration of 10 μM. Fluorescence was measured every 30 s for 4 min using the SpectraMax M2 microplate reader (Ex: 350 nm; Em: 420 nm) (Molecular Devices, San Jose, USA). Increasing concentrations of colistin were then added to designated wells, and the fluorescence intensity was measured every 30 s for an additional 20 min. Three technical replicates and three biological replicates were performed for each isolate and strain; averages and standard deviations were calculated, and a pairwise *t*-test (*P* < 0.05) was used to determine any significant differences. Fold change was also determined (colistin treated versus untreated for each pair).

### Biofilm formation

A biofilm assay was performed according to Sykes et al. (8). In brief, overnight cultures of all four *A. seifertii* isolates, as well as *A. baumannii* ATCC 17978-VU and *A. seifertii* DSM 102854,^T^ were standardized to an OD_600_ of 1.0 and aliquoted into 96-well flat bottom polystyrene plates. The samples were incubated at 37°C for 48 h. The OD_600_ was measured before removal of planktonic cells and biofilm staining with 0.1% crystal violet. The stained biofilm was solubilized using 30% acetic acid, and the crystal violet stain was measured at 550 nm. Biofilm formation was calculated relative to growth (OD_550_/OD_600_). Five technical replicates and at least three biological replicates were performed for each isolate and strain. One-way ANOVA with Dunnett’s post-hoc analysis was used in GraphPad Prism v 10.0.0 to determine significance between *A. baumannii* ATCC 17978-VU and the *A. seifertii* isolates. A pairwise *t*-test (*P* < 0.05) was used to determine any significant differences between the *A. seifertii* isolates.

### Surface-associated motility

Surface-associated motility of IK22, IK23, IK24, and IK25 was compared with that of *A. baumannii* ATCC 17978-VU and *A. seifertii* DSM 102854^T^ based on a protocol from Sykes et al. (8). In short, overnight cultures of each isolate and strain were standardized to an OD_600_ of 1.0. Three microliters of each sample was stab-inoculated into the center of semi-solid minimal media and incubated at 37°C for 18 h. The diameter of the distance traveled was measured from three different points. Three technical replicates and at least three biological replicates were performed for each isolate and strain. One-way ANOVA with Dunnett’s *post-hoc* analysis was used in GraphPad Prism v 10.0.0 to determine significance between *A. baumannii* ATCC 17978-VU and the *A. seifertii* isolates. A pairwise *t*-test (*P* < 0.05) was used to determine any significant differences between the *A. seifertii* isolates.

### Virulence in *Galleria mellonella*

The *G. mellonella* (waxworm) insect infection model was used to assess the virulence of the *A. seifertii* isolates using a protocol described in Sykes et al. (8). Briefly, overnight cultures of the four isolates, *A. baumannii* ATCC 17978-VU, and *A. seifertii* DSM 102854^T^ were standardized to 0.5 MacFarland in 1× PBS before 10 µL was injected into the left hind proleg of sets of 10 larvae. A set of controls (no injection, PBS only) was also included. The waxworms were incubated at 37°C for 72 h with survival being monitored every 12 h. Two biological replicates with one set each were performed for each isolate and strain. Percent end-point survival at 72 h was assessed. A pairwise *t*-test (*P* < 0.05) was used to determine any significant differences compared to *A. seifertii* DSM 102854^T^.

## RESULTS

### *Acinetobacter* isolates from tank milk in Indonesia were identified as *A. seifertii*

Four *Acinetobacter* isolates were selectively isolated from tank milk in Bogor, Indonesia. Whole-genome sequencing, followed by ANIb and dDDH, was used to identify the isolates (Table 1). Each of the isolates had an ANIb value of >96% relative to *A. seifertii* UBA3035 (GCA_002367535.1) and *A. seifertii* DSM 102854^T^ (GCF_000368065.1) and <95% (the cut-off value for species identification) (34) relative to two strains of the more common ACB complex member, *A. baumannii* (ATCC 17978-VU) (NZ_CP018664.1) and AB030 (SAMN26199121). Values for dDDH between the isolates and the two *A. seifertii* reference strains ranged from 75 to 78% compared to a range of only 55 to 64%, with the two *A. baumannii* reference strains well below the 75% threshold (34). ANIb analysis was also used to define intra-species units (54–56), where an ANI value of 99.9% is the threshold to define strains using whole-genome sequences (55, 56). Comparing the maximum value for all ANI comparisons of the four isolates with this threshold, it appeared that there were two strains, that is, one comprising IK22, IK23, and IK24 and the other with IK25 only; however, the four isolates are very closely related as the minimum ANI value between them was 99.80%. This close relationship was also evident, as the four new isolates were assigned to the novel sequence type ST-2706 using the Pasteur MLST scheme. A clonal complex could not be assigned through goeBURST.

With high confidence, based on these analyses, our tank milk isolates were identified as *A. seifertii*.

**TABLE 1:** Average nucleotide identity by BLAST and digital DNA-DNA hybridization comparison of the *A. seifertii* isolates (IK22, IK23, IK24, and IK25) with *A. seifertii* strains^*a*^^,*b*^

Isolate/strain	IK22	IK23	IK24	IK25	*A. seifertii* UBA3035	*A. seifertii* DSM 102854^T^	*A. baumannii* AB030	*A. baumannii* ATCC 17978-VU
IK22	100 (100)	99.95 (99.9)	100 (100)	99.81 (96.6)	96.56 (76.2)	96.86 (74.9)	89.41 (55.7)	89.37 (62.3)
IK23	99.99 (99.9)	100 (100)	99.99 (99.9)	99.80 (96.0)	96.56 (76.6)	96.86 (75.0)	89.39 (56.2)	89.32 (62.8)
IK24	100 (100)	99.96 (99.9)	100 (100)	99.82 (96.5)	96.56 (76.1)	96.90 (74.8)	89.41 (55.7)	89.35 (62.3)
IK25	99.82 (96.6)	99.80 (96.0)	99.82 (96.5)	100 (100)	96.50 (77.5)	96.84 (74.7)	89.38 (56.9)	89.33 (63.0)
*A. seifertii* UBA3035	96.30 (76.2)	96.29 (76.6)	96.30 (76.1)	96.32 (77.5)	100 (100)	96.71 (81.9)	89.29 (60.1)	89.15 (63.8)
*A. seifertii* DSM 102854^T^	96.48 (74.9)	96.47 (75.0)	96.48 (74.8)	96.61 (74.7)	96.76 (81.9)	100 (100)	89.40 (57.1)	89.30 (62.6)
*A. baumannii* AB030	89.32 (55.7)	89.32 (56.2)	89.31 (55.7)	89.11 (56.9)	89.29 (60.1)	89.39 (57.1)	100 (100)	96.91 (73.8)
*A. baumannii* ATCC 17978-VU	89.46 (62.3)	89.46 (62.8)	89.46 (62.3)	89.46 (63.0)	89.43 (63.8)	89.62 (62.6)	97.33 (73.8)	100 (100)

^*a*^ Digital DNA:DNA hybridization values are in parentheses.

^*b*^ The *A. baumannii* ATCC 17978-VU reference strain and *A. baumannii* AB030 were used as out-of-species comparators.

### *A. seifertii* isolates displayed colistin resistance

The four *A. seifertii* isolates were subjected to a Canadian study-based panel of antibiotics (CANWARD) (41) to determine their antibiotic susceptibility profiles. As there are no clinical breakpoints for *A. seifertii* specifically, CLSI breakpoints for *Acinetobacter* species were applied (42). Notably, all four isolates were resistant to colistin with MIC values two to fourfold higher than the ≥4 µg/mL breakpoint; the *A. seifertii* type strain and *A. baumannii* reference strain showed intermediate resistance to colistin (Table 2). Growth curves (Fig. S1) of the four isolates showed near-equal growth through exponential phase, with three isolates (IK22, IK23, IK24) making slower progress through stationary phase compared with *A. seifertii* DSM 102854^T^ and *A. baumannii* ATCC 17978-VU. Such slowed growth due to the fitness cost of colistin resistance has been observed previously (57).

**TABLE 2:** Antibiotic susceptibility profiles of the *A. seifertii* isolates (IK22, IK23, IK24, and IK25)^*b*^^,^^*c*^

Strain/isolate	ATCC 17978^*a*^	DSM 102854^T*a*^	IK22	IK23	IK24	IK25
AMK	0.5	1	0.5	0.5	1	0.5
CFZ	>128	>128	**>**128	>128	>128	>128
FEP	4	4	4	2	4	4
FOX	>32	>32	>32	>32	>32	>32
CAZ	8	16	16	16	16	**32**
BPR	0.5	1	0.5	0.5	0.5	1
C/T	2	0.5	1	2	2	4
CRO	16	16	32	16	16	16
CIP	0.25	0.12	0.25	0.25	0.5	0.5
CLR	32	8	8	16	8	16
CLI	>8	>8	>8	>8	>8	>8
CST	2	2	**8**	**16**	**16**	**8**
DAP	>16	>16	**>**16	>16	>16	>16
DOX	0.25	0.25	≤0.12	≤0.12	0.25	0.25
ETP	4	16	4	8	4	8
GEN	1	<0.5	2	2	2	4
IPM	0.25	0.25	0.25	0.5	0.25	0.25
LZD	>16	>16	>16	>16	>16	>16
MEM	0.5	1	0.25	0.5	0.25	0.5
NIT	>512	512	256	128	256	512
TZP	4	2	8	4	8	8
TOB	1	<0.5	1	1	1	1
SXT	4	≤0.12	≤0.12	≤0.12	≤0.12	≤0.12
VAN	>32	>32	>32	>32	>32	>32

^*a*^ *A. baumannii* ATCC 17978-VU; *A. seifertii* DSM 102854^T^.

^*b*^ AMK, Amikacin; CFZ, Cefazolin; FEP, Cefepime; FOX, Cefoxitin; CAZ, Ceftazidime; BPR, Ceftobiprole; C/T, Ceftolazane/tazobactam; CRO, Ceftriaxone; CIP, Ciprofloxacin; CLR, Clarithromycin; CLI, Clindamycin; CST, Colistin; DAP, Daptomycin; DOX, Doxycycline; ETP, Ertapenem; GEN, Gentamicin; IPM, Imipenem; LZD, Linezolid; MEM, Meropenem; NIT, Nitrofurantoin; TZP, Piperacillin/Tazobactam; TOB, Tobramycin; SXT, Trimethoprim/Sulphamethoxazole; VAN, Vancomycin.

^*c*^ The minimum inhibitory concentration is shown in µg/mL. Values that indicate resistance according to CLSI guidelines for *Acinetobacter* species are highlighted in bold; values that indicate intermediate susceptibility are underlined. The *A. baumannii *ATCC 17978-VU reference strain and *A. seifertii *DSM 102854^T^ type strain were included as comparators.

### The means of colistin resistance was not a known mechanism

**Fig 1:**
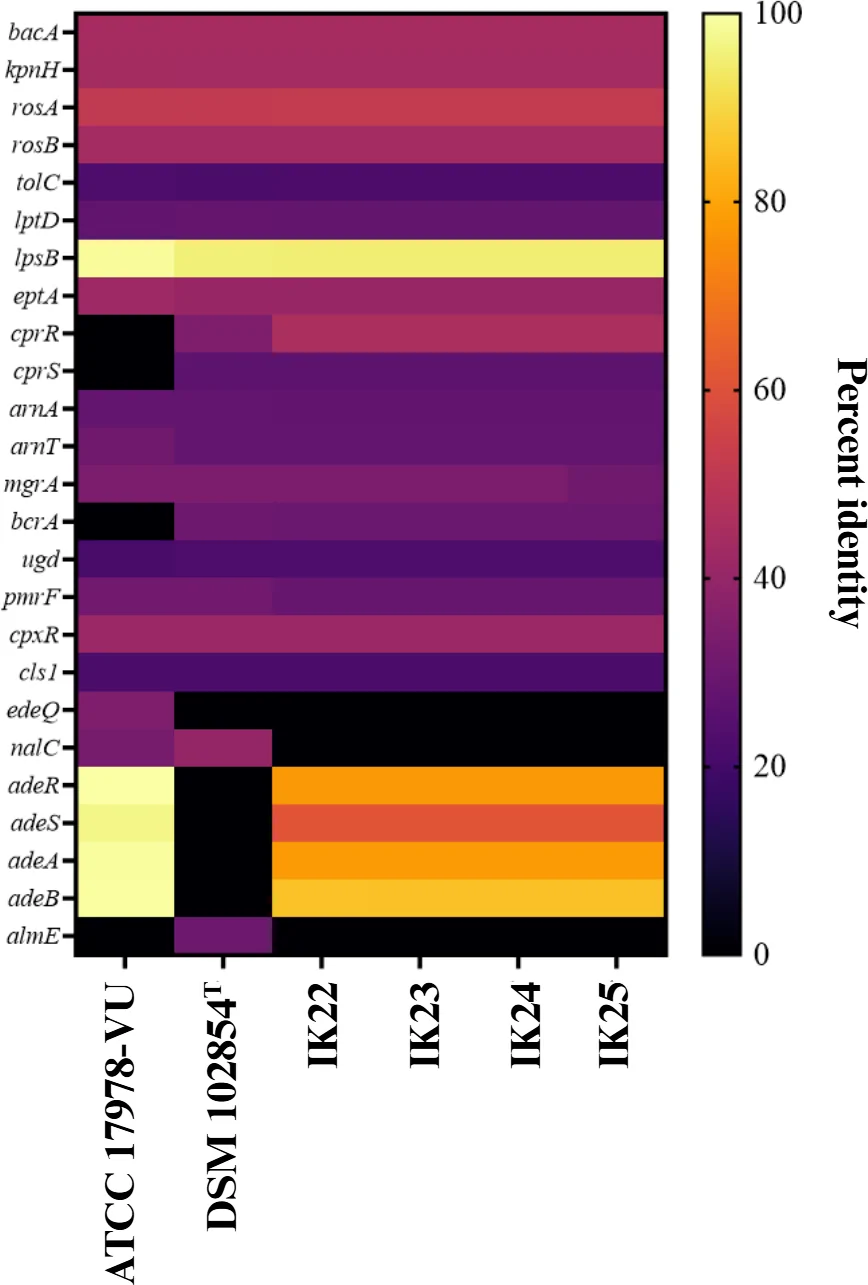
Colistin resistome assessment. Colistin resistome of the *A. seifertii* isolates (IK22, IK23, IK24, and IK25) compared with *A. seifertii* type strain DSM 102854^T^ and *A. baumannii* ATCC 17978-VU. Genes associated with colistin resistance from the comprehensive antibiotic resistance database Resistance Gene Identifier (perfect, strict, and loose hits) are shown on the left with percent identity indicated by a color scale on the right.

To understand the mechanism of colistin resistance of the four *A. seifertii* isolates, the RGI from the CARD was employed (43). Perfect and strict parameters yielded no known colistin resistance-related genes; however, loose parameters identified some relevant gene similarities and differences between colistin-susceptible and resistant strains (Fig. 1). Of note is the conservation of *lpsB* (which is involved in LOS synthesis) (58) with 98.9, 95.6, and 95.1% identity for *A. baumannii* ATCC 17978-VU, *A. seifertii* DSM 102854^T^, and the four isolates, respectively. The gene for phosphoethanolamine transferase, *eptA* (which modifies lipid A) (59), was detected with near-equal identity (~41%) among the isolates. A glaring difference between the *A. seifertii* reference strain and the tank milk isolates was the presence of the resistance-nodulation-division (RND) efflux pump genes *adeAB* and its regulator *adeRS*. This observation is of interest as these genes were the only *Acinetobacter* species-specific genes in the RGI output and are linked with antibiotic resistance (though not colistin specifically) (60). Using RT-qPCR, the expression of *adeB* (as a proxy for the entire operon) was found to be significantly lower in the *A. seifertii* isolates versus *A. baumannii* ATCC 17978-VU (Fig. S2).

Most of the genes detected in the RGI analysis were not unique to *Acinetobacter* species, and a literature search of each gene in relation to *A. baumannii* colistin resistance yielded no further information to guide us toward a mechanism of resistance. Using TBLASTN (44), the products of the gene sequences observed in the CARD results for each *A. seifertii* isolate and the two reference strains (*A. baumannii* ATCC 17978-VU and *A. seifertii* DSM 102854^T^) were then manually compared across each genome. Though amino acid substitutions were observed in 12 (Fig. S3 through S14) of the 25 proteins, most were uncharacterized with respect to colistin resistance.

### Lipidomic analysis revealed two compounds in greater abundance in the *A. seifertii* isolates

**Fig 2:**
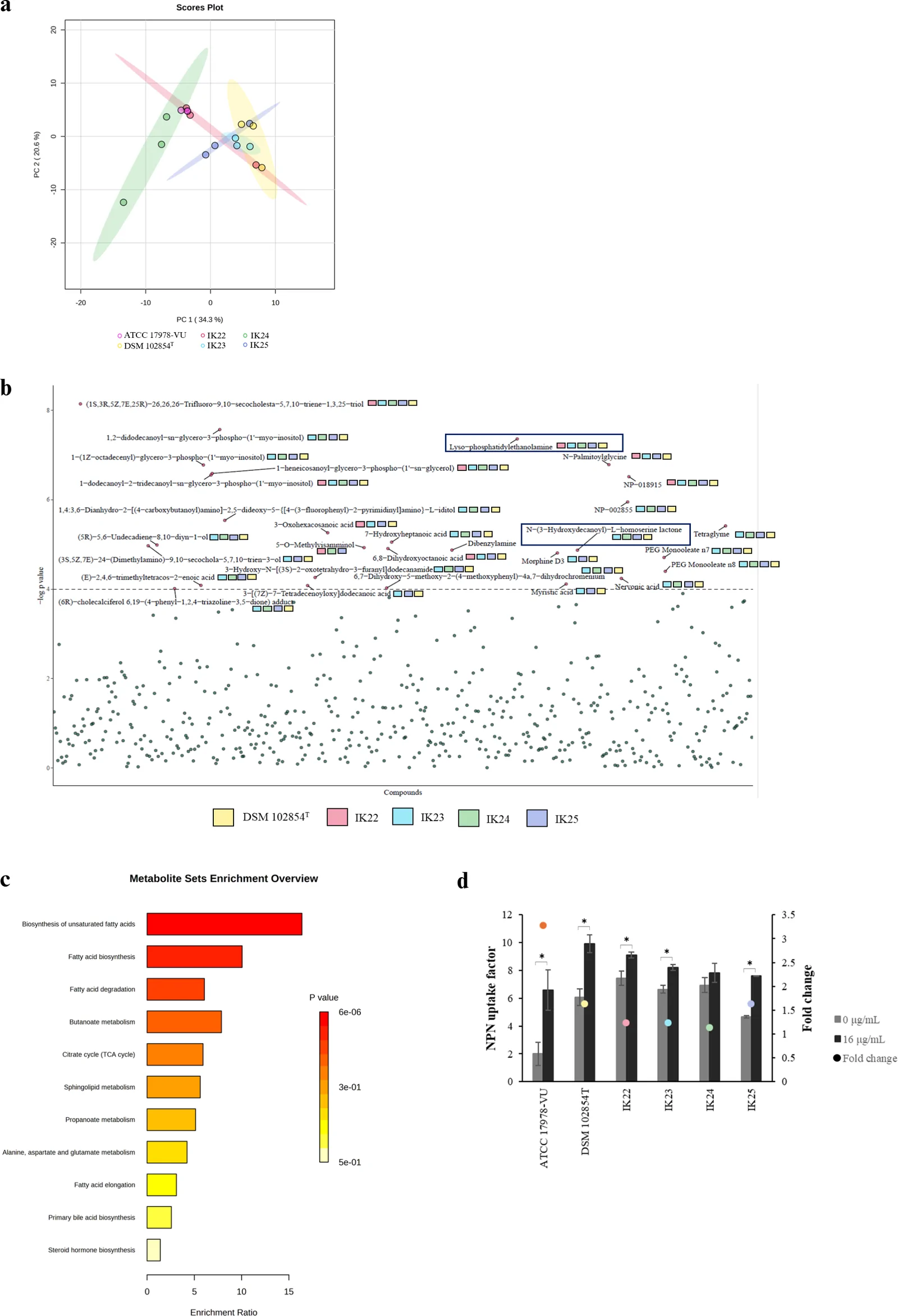
Lipidomic analysis. (**a**) Principal component analysis of the variance between the *A. seifertii* isolates (IK22, IK23, IK24, and IK25), the *A. seifertii* type strain DSM 102854^T^, and *A. baumannii* ATCC 17978-VU (95% CI; *n* = 3 biological replicates). (**b**) Global lipid profile of the *A. seifertii* isolates compared to *A. baumannii* ATCC 17978-VU. Compounds of statistical significance (one-way ANOVA; ****P ≤ 0.0001) are above the dotted line. Colored squares indicate the *A. seifertii* isolate that showed that compound in greater abundance than *A. baumannii* ATCC 17978-VU. Lyso-phosphatidylethanolamine (lyso-PE) and N-(3-hydroxydecanoyl)-L-homoserine lactone are highlighted with blue boxes to indicate their importance. (**c**) Enrichment analysis of the lipidomic data from the *A. seifertii* isolates using KEGG pathway categories. (**d**) Membrane integrity of the *A. seifertii* isolates assessed using 1-N-phenylnaphthylamine (NPN). NPN uptake was determined for all isolates in the absence (0 µg/mL) and presence (16 µg/mL) of colistin (pairwise *t*-test; *P < 0.05; *n* = 2–6 biological replicates), and the fold change was calculated (represented by the colored circles).

Given that colistin interacts with the lipid A component of the bacterial outer membrane (19), total lipids extracted from each *A. seifertii* isolate were analyzed via liquid chromatography–mass spectrometry. Principal component analysis was applied to evaluate any variance between the colistin-resistant and -sensitive strains (Fig. 2a). *A. seifertii* isolate IK22 clustered tightly with colistin-sensitive *A. baumannii* ATCC 17978-VU; IK23 clustered most closely with colistin-sensitive *A. seifertii* DSM 102854^T^; and IK24 and IK25 clustered separately. Lipidome metabolite analysis identified 559 compounds, with 30 found to be significantly different (*P* ≤ 0.0001) between *A. baumannii* ATCC 17978-VU and the four isolates combined (Fig. 2b). Metabolic enrichment analysis based on KEGG pathways revealed that the most highly enriched lipids are involved in the biosynthesis of unsaturated fatty acids and the synthesis and degradation of fatty acids (Fig. 2c). Though there was no detection of phosphoethanolamine modification of lipid A as anticipated, two differentially abundant compounds were of particular interest: lyso-phosphatidylethanolamine (lyso-PE) and N-(3-hydroxydecanoyl)-L-homoserine lactone (3-OH-C10 HSL) (Fig. 2b).

Lyso-PE is a known component of *A. baumannii* membranes, and higher abundance of such lyso-glycerophospholipids has been linked to membrane remodeling due to loss of LOS, thereby facilitating colistin resistance (19). However, the LOS has not been lost in these *A. seifertii* isolates (Fig. S15). Furthermore, a membrane permeabilization assay using NPN (53) generally showed a marked fluorescence intensity increase in colistin-treated cells versus untreated cells, suggesting that colistin was able to interact with the LOS, disrupt the outer membrane, and allow NPN to access the hydrophobic inner membrane (Fig. 2d), although the fold change was <2, and a concentration-dependent response was not observed (Fig. S16).

**Fig 3:**
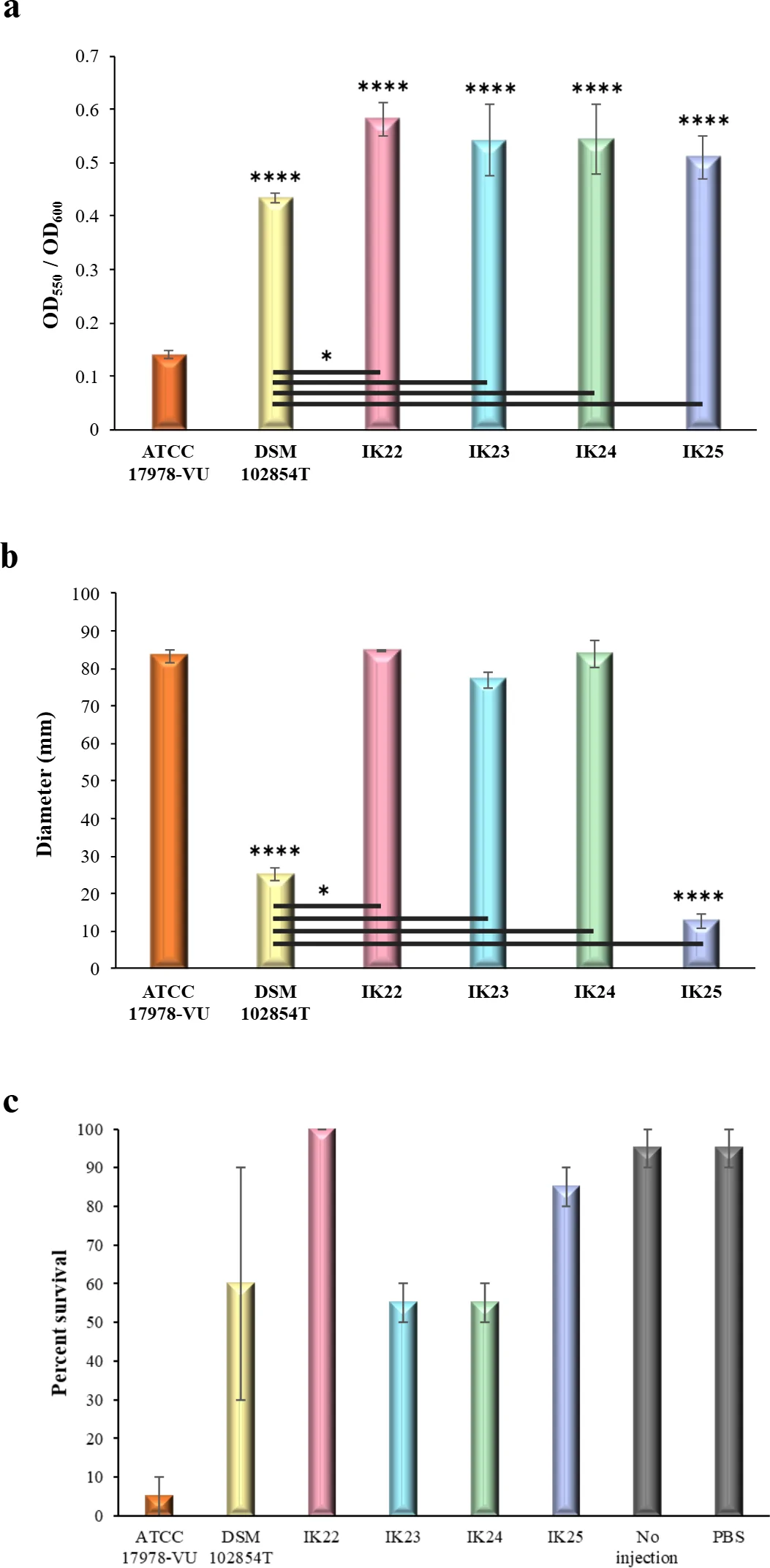
Phenotypic characterization. (**a**) Biofilm formation of the *A. seifertii* isolates (IK22, IK23, IK24, and IK25) compared with the *A. seifertii* type strain DSM 102854^T^ and *A. baumannii* ATCC 17978-VU. Biofilm was determined via crystal violet staining (550 nm) and normalized to growth (600 nm). Isolates with significant difference from *A. baumannii* ATCC 17978-VU are indicated with asterisks (one-way ANOVA; ****P < 0.0001); *A. seifertii* isolates with significant difference from *A. seifertii* DSM 102854^T^ are indicated with lines and a single asterisk (pairwise *t*-test; *P < 0.05) (*n* = 3 biological replicates each with 5 technical replicates). (**b**) Motility of the *A. seifertii* isolates compared with *A. seifertii* DSM 102854^T^ and *A. baumannii* ATCC 17978-VU. The diameter (mm) of the distance traveled on semi-solid media was measured after 18 h of incubation at 37°C. Isolates with significant difference from *A. baumannii* ATCC 17978-VU are indicated with asterisks (one-way ANOVA; ****P < 0.0001); *A. seifertii* isolates with significant difference from *A. seifertii* DSM 102854^T^ are indicated with brackets and a single asterisk (pairwise *t*-test; *P < 0.05) (*n* = ≥3 biological replicates each with ≥3 technical replicates). (**c**) Virulence of the *A. seifertii* isolates in the *Galleria mellonella* insect infection model. Percent survival of injected larvae was assessed after 72 h at 37°C. No significant differences were observed between the *A. seifertii* isolates (pairwise *t*-test; *P* < 0.05; *n* = 2 biological replicates each with a set of 10 waxworms). *A. baumannii* ATCC 17978-VU was included as a known virulent strain (8).

A member of the N-acyl homoserine lactone (AHL) family of compounds involved in quorum sensing, 3-OH-C10 HSL (61), was also significantly more abundant in *A. seifertii* DSM 102854^T^, IK23, IK24, and IK25 than in *A. baumannii* ATCC 17978-VU. This observation led us to assay quorum sensing-mediated phenotypes (biofilm formation, motility) and virulence (insect infection model). The tank milk *A. seifertii* isolates showed biofilm formation equal to *A. seifertii* DSM 102854^T^ (Fig. 3a), and three isolates (IK22, IK23, and IK24) had ~4 fold increased surface-associated motility (Fig. 3b). The well-documented *G. mellonella* (waxworm) insect infection model (62) was used to assess the virulence of the *A. seifertii* isolates and found all four to be less or equally virulent (but not significantly different) than *A. seifertii* DSM 102854^T^ up to 72 h (Fig. 3c).

## DISCUSSION

Colistin is considered a last-resort antibiotic in clinical settings due to its cytotoxicity, but it is more generally used in agricultural and veterinary practices as a prophylactic antibiotic (16). Our discovery of four *A. seifertii* isolates that possess colistin resistance from tank milk in Indonesia exemplifies the increasing prevalence of colistin-resistant bacteria across varying geographies, bacterial species, and sample types (63). The fact that our isolates are non-clinical is intriguing and adds to the tally of colistin-resistant (12, 15) and colistin-susceptible isolates (64).

Resistance to colistin in *A. baumannii* is facilitated by multiple determinants, including plasmid-mediated mobile colistin resistance genes (24–26) and disruption of LOS biosynthesis genes by insertion elements (27), but little is known about the molecular resistance mechanisms in *A. seifertii*. Prediction of the resistomes of the four tank milk isolates using the CARD revealed no such known determinants of colistin resistance (Fig. 1), although we did observe two interesting genetic differences between *A. baumannii* ATCC 17978-VU, the *A. seifertii* reference strain DSM 102854^T^, and the four tank milk isolates that may contribute to it. First, we observed the presence of the RND efflux pump operon *adeAB* in our isolates but not in the *A. seifertii* reference strain (Fig. 1). The abundance of the *adeB* transcript was found to be low relative to *A. baumannii* ATCC 17978-VU (Fig. S2), but the simple presence of AdeAB may be contributing to the colistin-resistance phenotype. Typically, increased expression of RND efflux pumps is linked to decreased susceptibility (increased resistance) because antibiotics are being removed by the pumps. Here, though, we know that reference strains *A. baumannii* ATCC 17978-VU and *A. seifertii* DSM 102854^T^ are sensitive to colistin (Table 2), despite the presence or absence, respectively, of AdeAB, so the exact role played by this pump in our isolates is unclear. Due to the biochemical structure of colistin, it is unlikely to be a direct substrate of the efflux pump, but an efflux-mediated colistin resistance mechanism is possible as a study by Yilmaz and colleagues (65) showed that a colistin-resistant *A. baumannii* strain became susceptible to colistin in the presence of the efflux pump inhibitor carbonyl cyanide 3-chlorophenylhydrazone (CCCP).

Second, we found mutations in AdeS (the transcriptional activator of AdeAB) (Fig. S12). Two mutations within AdeS that have previously been suggested to play a role in the colistin-resistance phenotype, namely, V27L and L172P (65), were present in each of our four *A. seifertii* isolates. These mutations may explain the seemingly low expression levels of *adeB* we observed relative to *A. baumannii* ATCC 17978-VU (Fig. S2); however, contrary to this idea, the same two AdeS mutations were detected in the colistin-sensitive reference strain, *A. seifertii* DSM 102854^T^, in the absence of the target operon *adeAB*. Elucidation of any link between *adeAB* expression and colistin resistance requires further study. Additionally, as colistin resistance has been linked to substitutions in the *lpxDCA* operon in *Acinetobacter colistiniresistens* (66), and as dysfunctional homologous LPS pathway proteins in *E. coli* have been observed to play a role in polymyxin resistance (67), the SNPs discovered in the *lpxDCA* operon of our *A. seifertii* isolates (Fig. S6 to S8) warrant investigation.

With minimal genetic clues to colistin resistance, we investigated the global lipidome of each of the *A. seifertii* isolates and compared them against *A. baumannii* ATCC 17978-VU and *A. seifertii* DSM 102854^T^ (Fig. 2b). Lipidomics, a subset of metabolomics, is a growing source of clues to bacterial pathogenicity (68), as the diversity of lipids and lipid derivatives that compose bacterial membranes of antibiotic-resistant and sensitive strains is being revealed (69–71). This analysis revealed many compounds with varying production between the four isolates, although identification was challenging because the bias of current databases toward eukaryotic lipids limits the number of metabolites that can be accurately interpreted in the bacterial context (68). Our study was also limited in that some compounds were not identified and were, therefore, part of the so-called ‘dark metabolome’ (72). We did detect two compounds of interest for the first time in *A. seifertii*. Lyso-PE has not yet been reported as a component of the *A. seifertii* membrane, and compared with *A. baumannii*, this glycerophospholipid was found in greater abundance. Also, this is the first identification of the quorum sensing molecule 3-OH-C10 HSL in *A. seifertii*. This molecule is linked with virulence and has been characterized in *A. baumannii* (61) and *A. nosocomialis* (73). The exact physiological contribution of this compound is yet to be determined, as the four tank milk *A. seifertii* isolates were not or minimally virulent in the insect infection model (Fig. 3c).

Other than colistin resistance, each of the *A. seifertii* isolates had a similar global lipid profile compared to the colistin-sensitive *A. seifertii* reference strain, with all compounds in higher abundance versus *A. baumannii* ATCC 17978-VU (Fig. 2b). This suggests that there is some fine-tuned control of other processes within the cell or alternative membrane modifications that were not detectable via our methods, the latter theory being supported by the subtle but otherwise static membrane permeability of the isolates in the presence of colistin (Fig. 2d and Fig. S16). Future investigation into the global transcriptomic and proteomic changes in these isolates may explain the little to no lipidomic differences between colistin-resistant and -susceptible *A. seifertii*.

To our knowledge, this study is the first to investigate *A. seifertii* isolates from Indonesia, though *Acinetobacter* species previously have been associated with milk (74, 75). Even though no known colistin-resistance mechanisms were detected in our isolates, and there was no history of colistin treatment of the dairy cows that produced the tank milk, it is possible that exposure to another polymyxin antibiotic could have triggered the observed colistin resistance and might be part of an unidentified mobile genetic element. Based on the information we gathered, we could not distinguish whether (i) the *A. seifertii* isolates harbor an unknown, novel resistance mechanism for colistin; (ii) the isolates were colistin tolerant (76); (iii) the isolates were heteroresistant (77); or (iv) we simply do not understand *A. seifertii* physiology well enough. We will continue to explore the diversity of non-human ACB complex members to gain a more comprehensive understanding of antibiotic resistance and discover new avenues of investigation for antimicrobial resistance prevention.

## Data Availability

NCBI GenBank Bioproject PRJNA819071; BioSample: AB348-IK22 (SAMN26898588), AB359-IK23 (SAMN26898589), AB349-IK24 (SAMN26898590), and AB350-IK25 (SAMN26898591); accession numbers: AB348-IK22 (JANBNW000000000), AB359-IK23 (JANBNX000000000), AB349-IK24 (JANBNY000000000), and AB350-IK25 (JANBNZ000000000); and MassIVE: MSV000103047.

## SUPPLEMENTAL MATERIAL

Supplemental figures spectrum.02311-26-s0001.pdf Figures S1 to S16.

ASM does not own the copyrights to Supplemental Material that may be linked to, or accessed through, an article. The authors have granted ASM a non-exclusive, world-wide license to publish the Supplemental Material files. Please contact the corresponding author directly for reuse.
