# Supplementary material for: Investigation of colistin-resistant non-human Acinetobacter seifertii isolates
Source: Microbiol Spectr. 2026 Sep 17;14(10):e02311-26. doi: 10.1128/spectrum.02311-26 (PMC13689285; doi:10.1128/spectrum.02311-26)
Supplement: Supplementary file 1 [file spectrum.02311-26-s0001.pdf]

Supplementary Information

Investigation of colistin-resistant non-human *Acinetobacter seifertii* isolates

Ellen M. E. Sykes[a], Yen Chi Dang[a], Dawn White[a], Valeria Mateo-Estrada[b], Anna Muzaleva[a], George Zhanel[c], Jeremy Dettman[d], Julie Chapados[d], Suzanne Gerdis[d], Izhar U. H. Khan[d], Santiago Castillo-Ramírez[b], Ayush Kumar[a#]

[a]Department of Microbiology, University of Manitoba, Winnipeg, Canada

[b]Programa de Genómica Evolutiva, Centro de Ciencias Génomicas, Universidad Nacional Autónoma de México, Cuernavaca, Mexico

[c]Department of Medical Microbiology and Infectious Diseases, University of Manitoba, Winnipeg, Canada

[d]Ottawa Research and Development Centre, Agriculture and Agri-Food Canada, Ottawa, Canada

Note: For simplicity, the *A. seifertii* isolate names have been shortened to IK22 (AB348-IK22), IK23 (AB359-IK23), IK24 (AB349-IK24), and IK25 (AB350-IK25) in all text, figure legends and labels.

Bacterial growth

The *A. seifertii* isolates were grown in Lysogeny Broth (LB) (BD, New Jersey, USA) at 37ºC for 18 hours with shaking. Subcultures (1:100) were created in fresh LB broth and 150 µL aliquots were transferred to the wells of a clear 96-well plate. Growth was monitored every 20 minutes for 24 hours at OD_{600} using a BioTek Synergy HTX plate reader (Agilent, Santa Clara, USA) held at 37ºC. Each curve represents the average of at least three biological replicates.

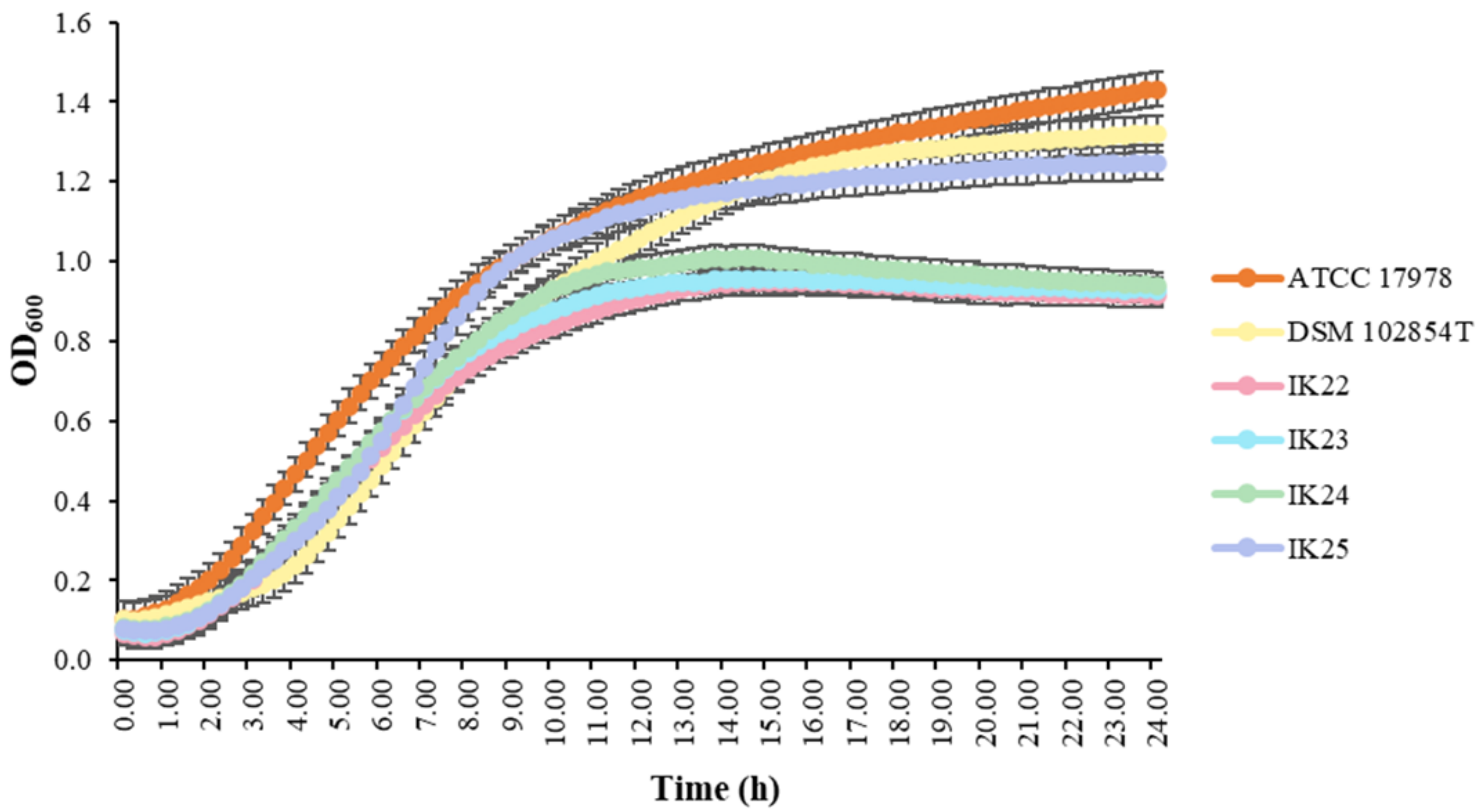

Fig. S1. Growth curves for the *A. seifertii* isolates (IK22, IK23, IK24, and IK25), type strain *A. seifertii* DSM 102854[T], and *A. baumannii* ATCC 17978-VU.

RND efflux expression evaluation

Expression analysis was performed according to Sykes et al [1]. In brief, mid-exponential phase cultures (OD_{600} of 0.7 ± 0.5) were pelleted and stored at -70ºC overnight. RNA was extracted using the Invitrogen Purelink RNA Extraction Kit, DNA was removed using the Purelink DNase Kit, and cDNA was synthesized using the Invitrogen VILO cDNA Kit (all kits from Thermo Fisher Scientific, Waltham, USA). The Applied Biosciences SYBR Select Master Mix (Beverly Hills, USA) was combined with the sample and primers (200 nM) and the reactions were run on an Applied Biosciences StepOnePlus system. RT-qPCR for each isolate was performed with three technical replicates and at least three biological replicates per target gene (*adeB* forward: 5′-GGATTATGGCGACTGAAGGA-3′; reverse: 5′-AATACTGCCGCCAATACCAG-3′: 16S rRNA forward: 5′-CTTCGGACCTTGCGCTAATA-3′; reverse: 5′-ATCCTCTCAGACCCGCTACA-3′). The Pfaffl method [2] of analysis was used to calculate the relative fold change expression compared to *A. baumannii* ATCC 17978-VU. One-way ANOVA using Dunnett's post-hoc analysis was performed using GraphPad Prism v 10.0.0.

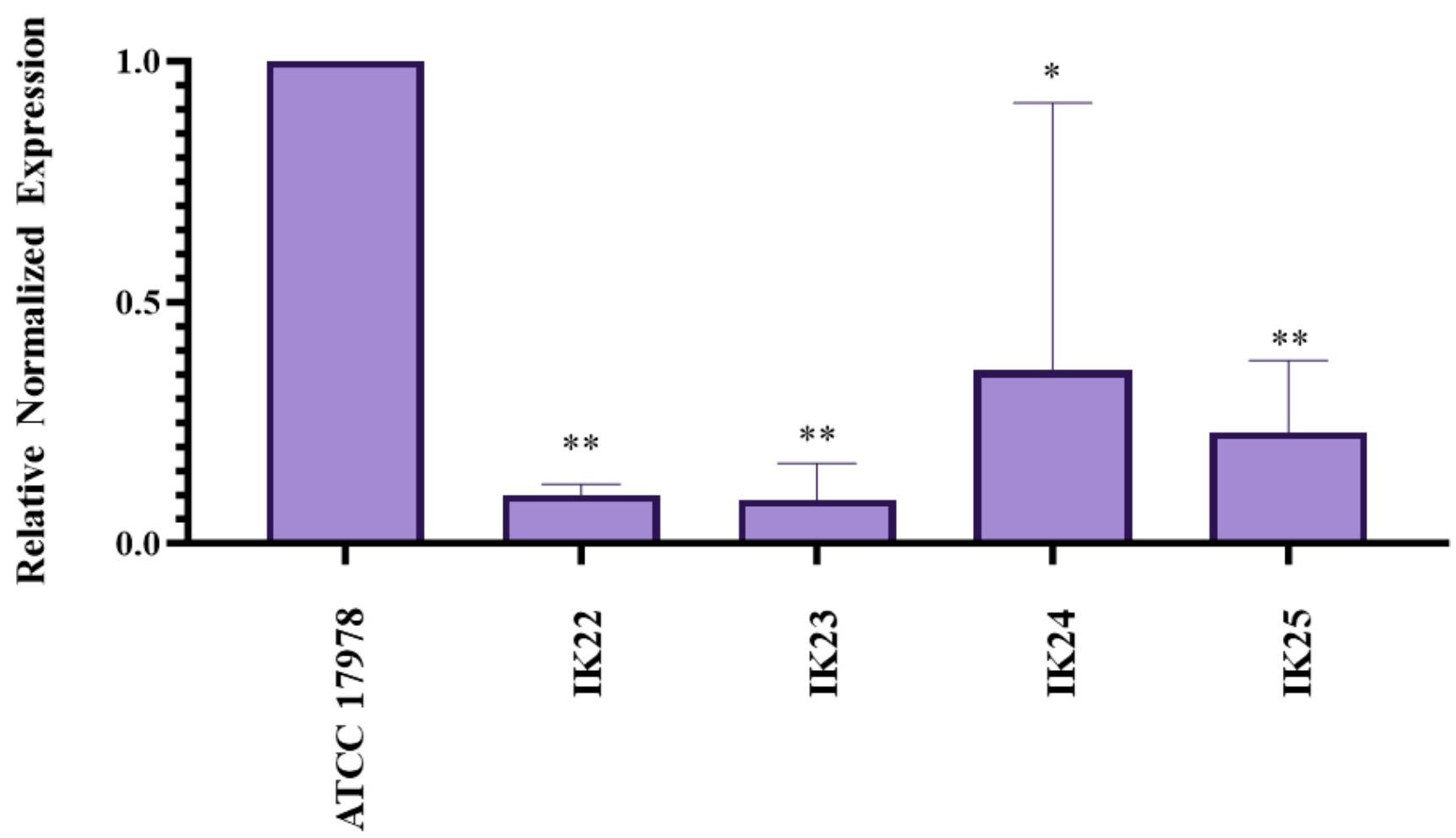

Fig. S2. Evaluation of *adeB* expression of the *A. seifertii* isolates by RT-qPCR. *A. baumannii* ATCC 17978-VU was used as the model for normalized expression (one-way ANOVA; * $p<0.1$, ** $p<0.01$; n = 3 biological replicates each with 3 technical replicates).

Alignment of colistin-resistance related genes

Comparison of known colistin resistance-related genes was conducted using NCBI tblastn [3]. Alignments were performed using MAFFT [4] and visualized using ESPript [5].

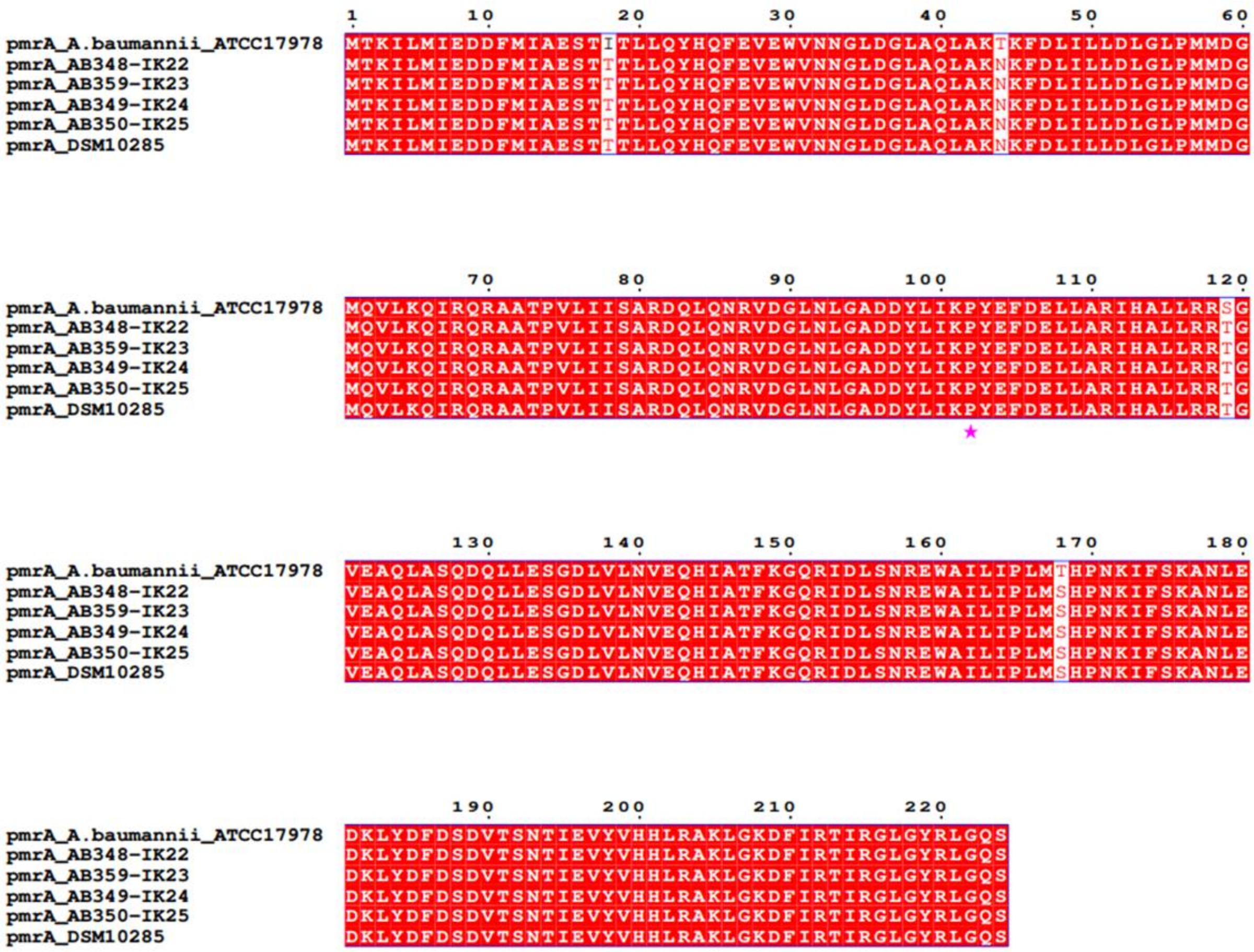

Fig. S3. Alignment of PmrA sequences from *A. baumannii* ATCC 17978-VU, *A. seifertii* DSM 102854^{T}, and the *A. seifertii* isolates (IK22, IK23, IK24, and IK25). The pink star indicates the location of a known mutation that confers colistin resistance in *A. baumannii* ATCC 17978-VU (P102H).

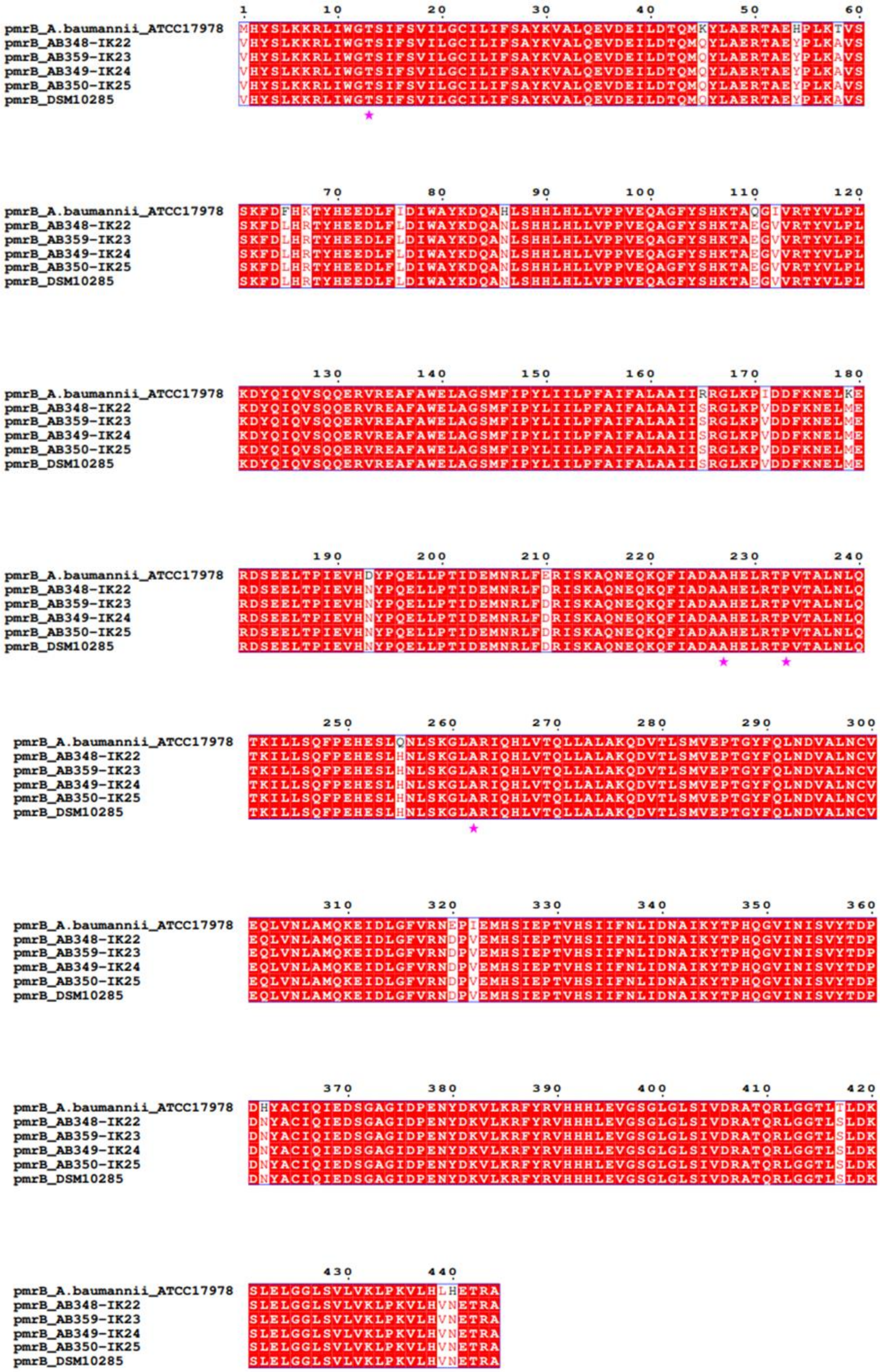
pmrB_A.baumannii_ATCC17978
pmrB_AB348-IK22
pmrB_AB359-IK23
pmrB_AB349-IK24
pmrB_AB350-IK25
pmrB_DSM10285

Fig. S4. Alignment of PmrB sequences from *A. baumannii* ATCC 17978-VU, *A. seifertii* DSM 102854^{T}, and the *A. seifertii* isolates (IK22, IK23, IK24, and IK25). The pink star indicates the location of a known mutation that confers colistin resistance in *A. baumannii* ATCC 17978-VU (T13N, A227V, P233T/S, A262P).

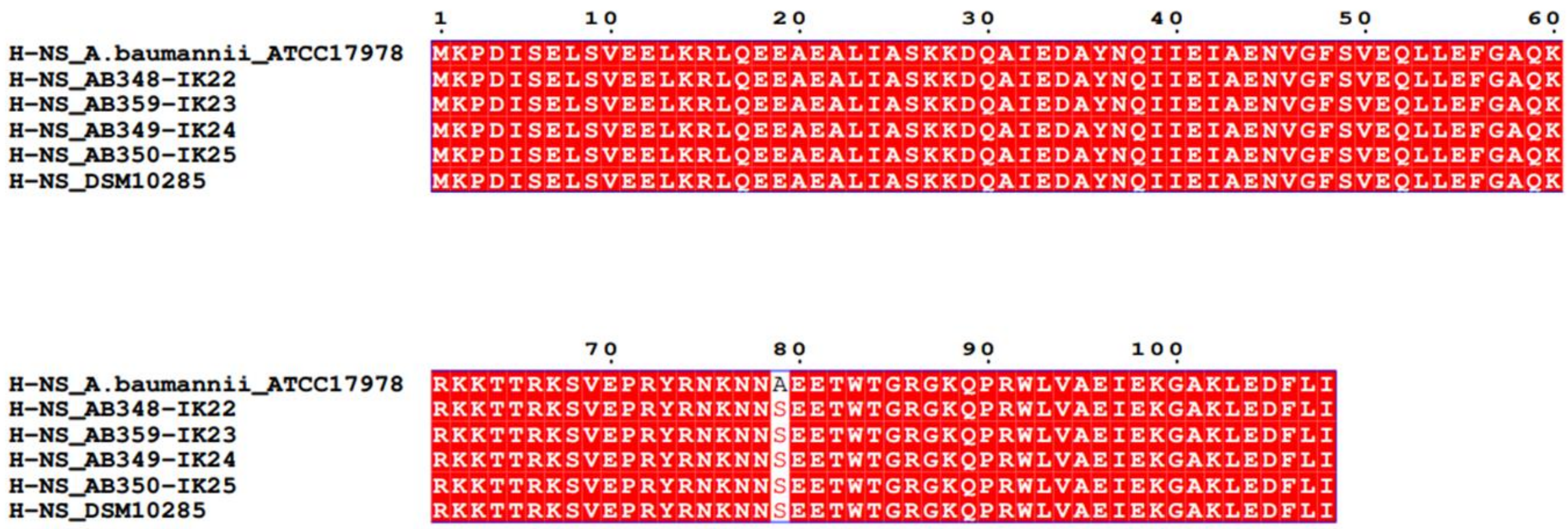

Fig. S5. Alignment of H-NS sequences from *A. baumannii* ATCC 17978-VU, *A. seifertii* DSM 102854^{T}, and the *A. seifertii* isolates (IK22, IK23, IK24, and IK25).

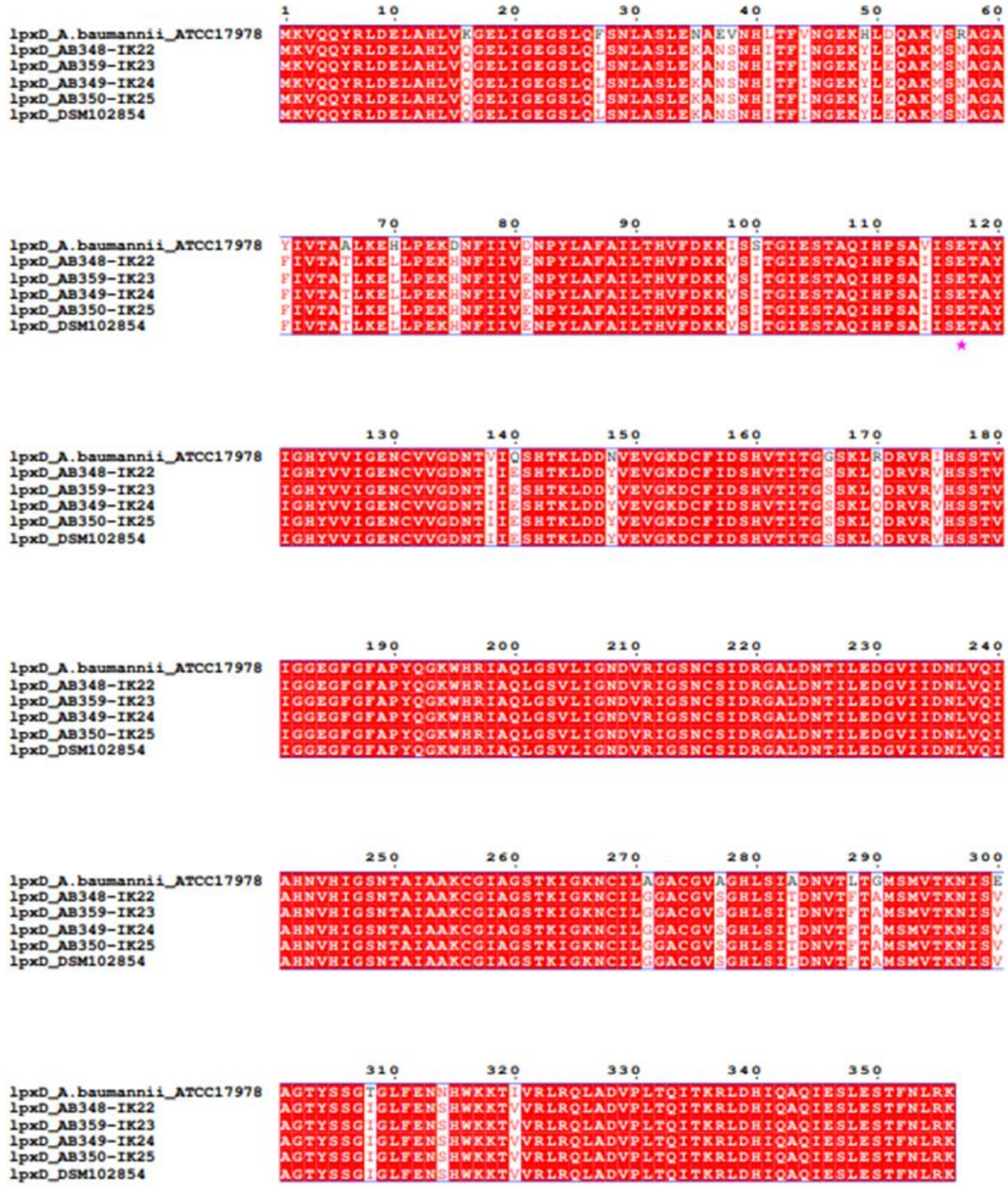

Fig. S6. Alignment of LpxD sequences from *A. baumannii* ATCC 17978-VU, *A. seifertii* DSM 102854^{T}, and the *A. seifertii* isolates (IK22, IK23, IK24, and IK25). The pink star indicates the location of a known mutation that confers colistin resistance in *A. baumannii* ATCC 17978-VU (E117K).

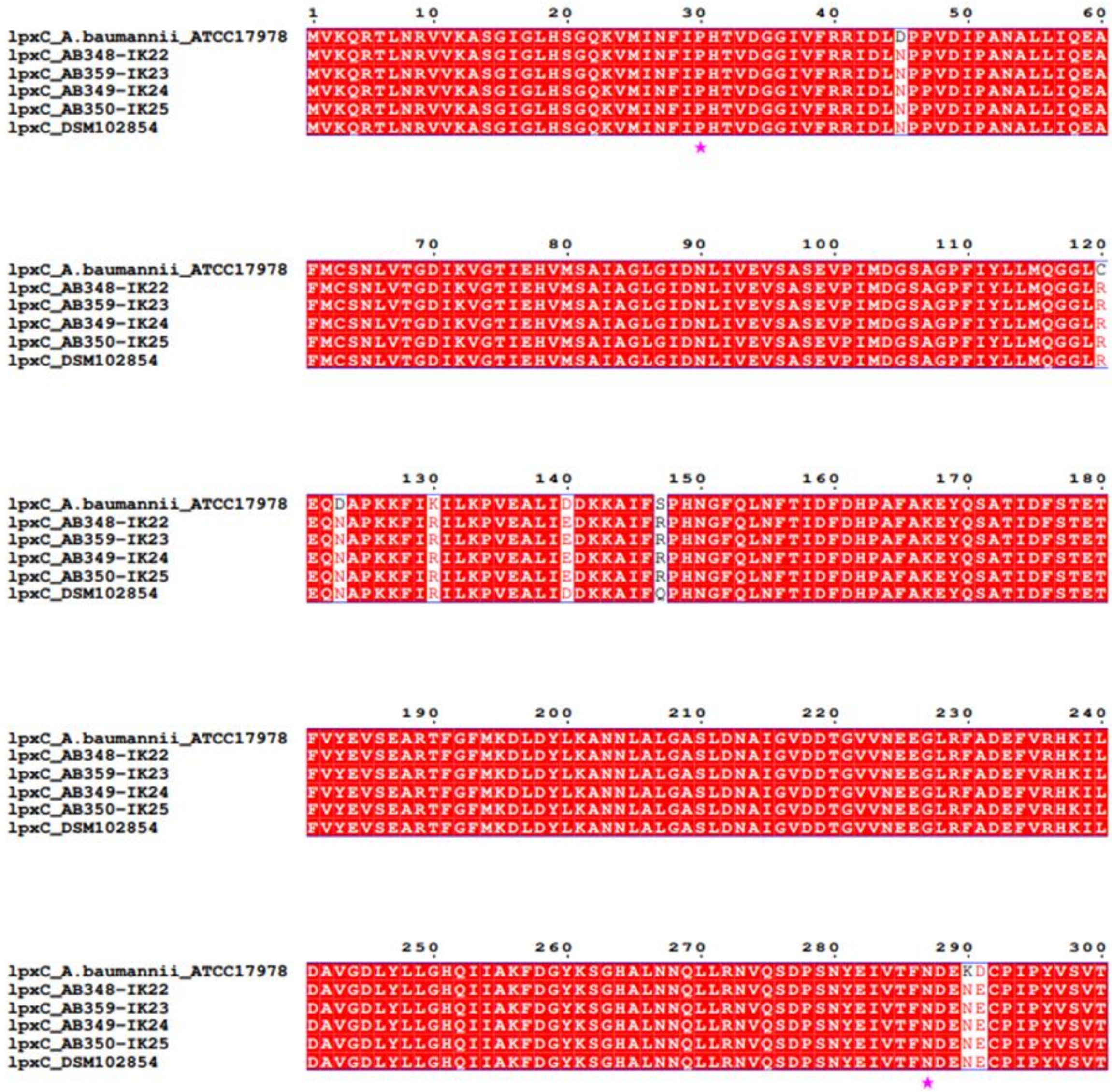

Fig. S7. Alignment of LpxC sequences from *A. baumannii* ATCC 17978-VU, *A. seifertii* DSM 102854^{T}, and the *A. seifertii* isolates (IK22, IK23, IK24, and IK25). The pink star indicates the location of a known mutation that confers colistin resistance in *A. baumannii* ATCC 17978-VU (P30L/S, N287D).

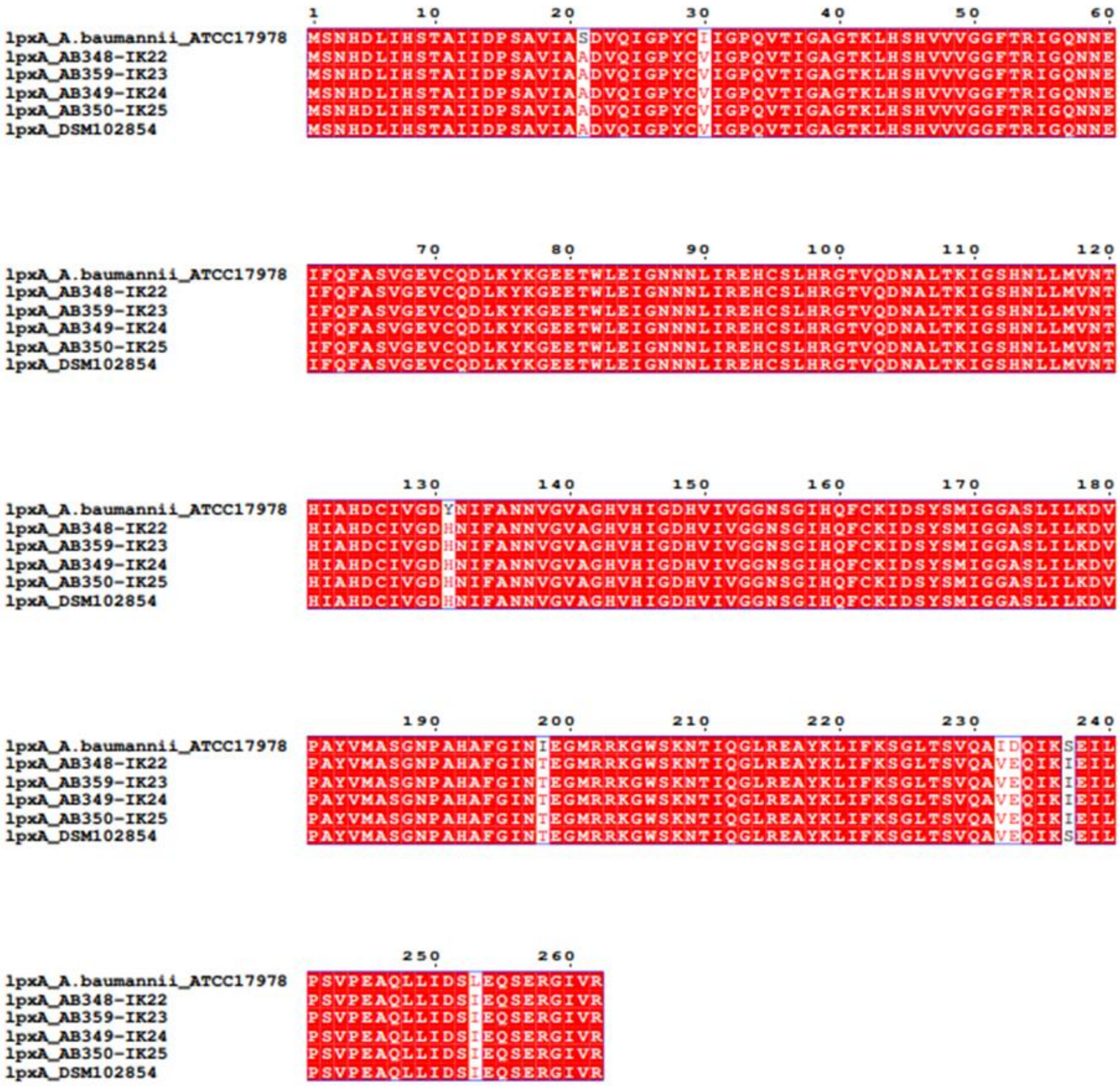

Fig. S8. Alignment of LpxA sequences from *A. baumannii* ATCC 17978-VU, *A. seifertii* DSM 102854^{T}, and the *A. seifertii* isolates (IK22, IK23, IK24, and IK25).

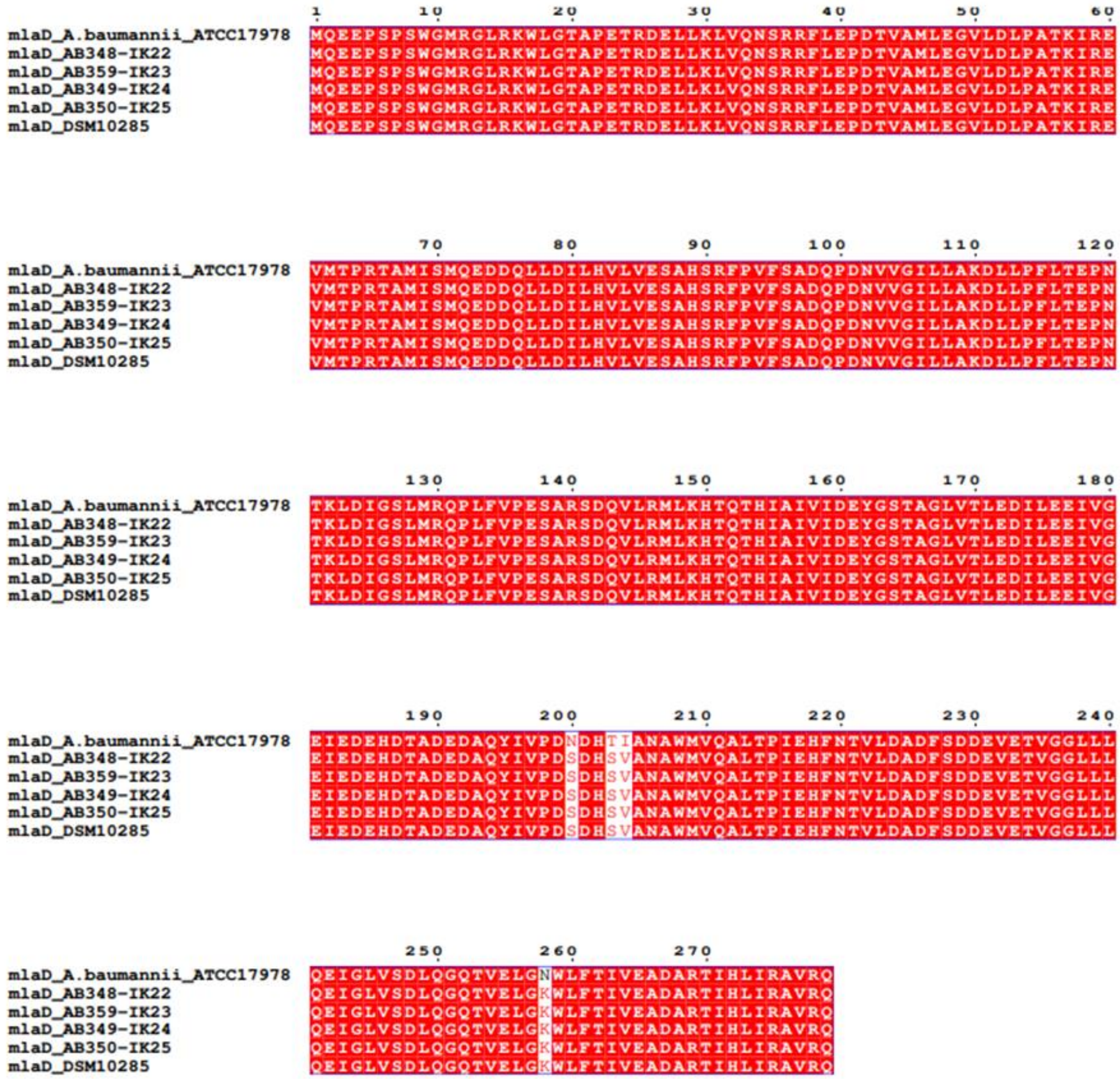

Fig. S9. Alignment of MlaD sequences from *A. baumannii* ATCC 17978-VU, *A. seifertii* DSM 102854^{T}, and the *A. seifertii* isolates (IK22, IK23, IK24, and IK25).

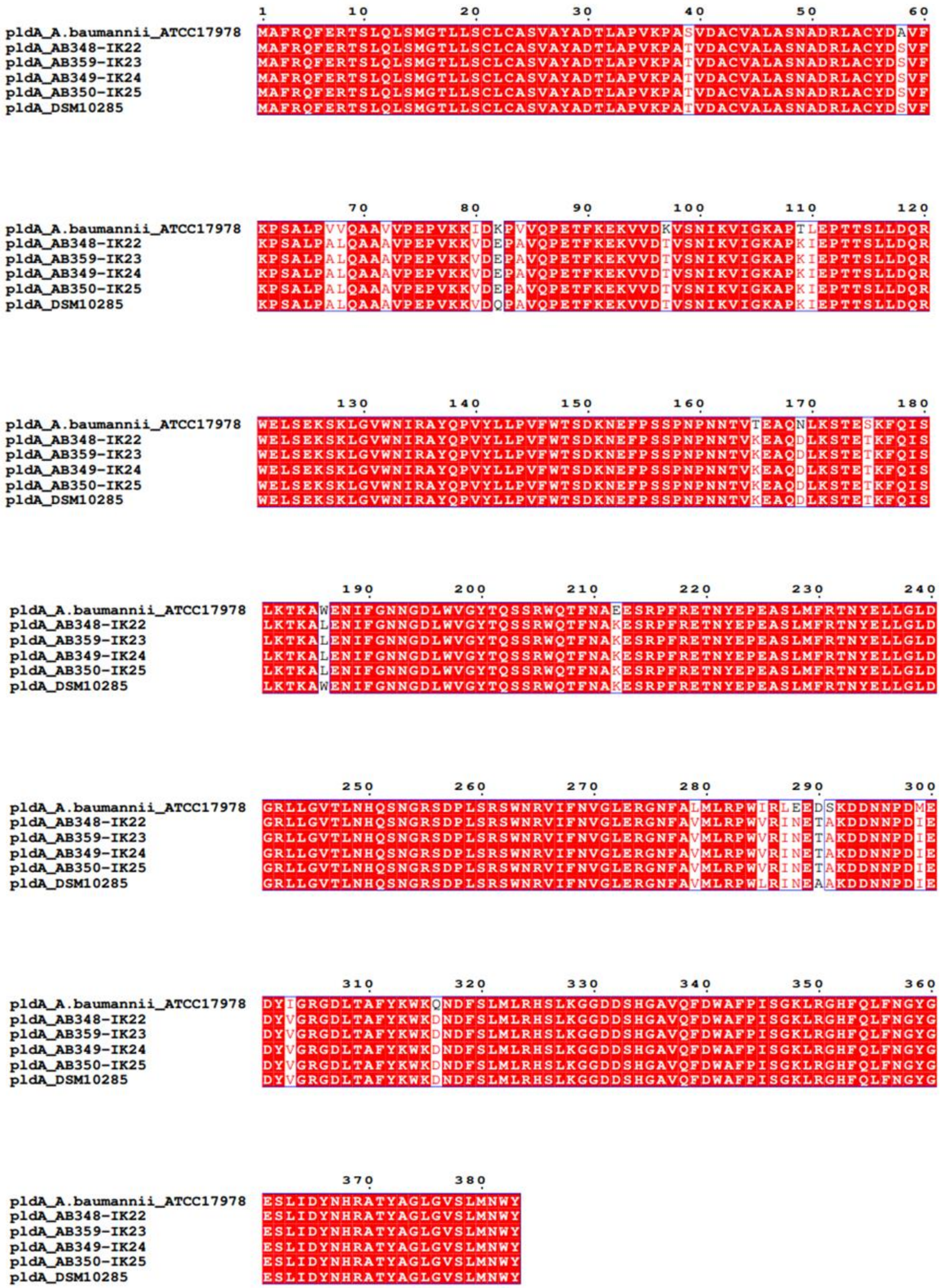

Fig. S10. Alignment of PldA sequences from *A. baumannii* ATCC 17978-VU, *A. seifertii* DSM 102854^{T}, and the *A. seifertii* isolates (IK22, IK23, IK24, and IK25).

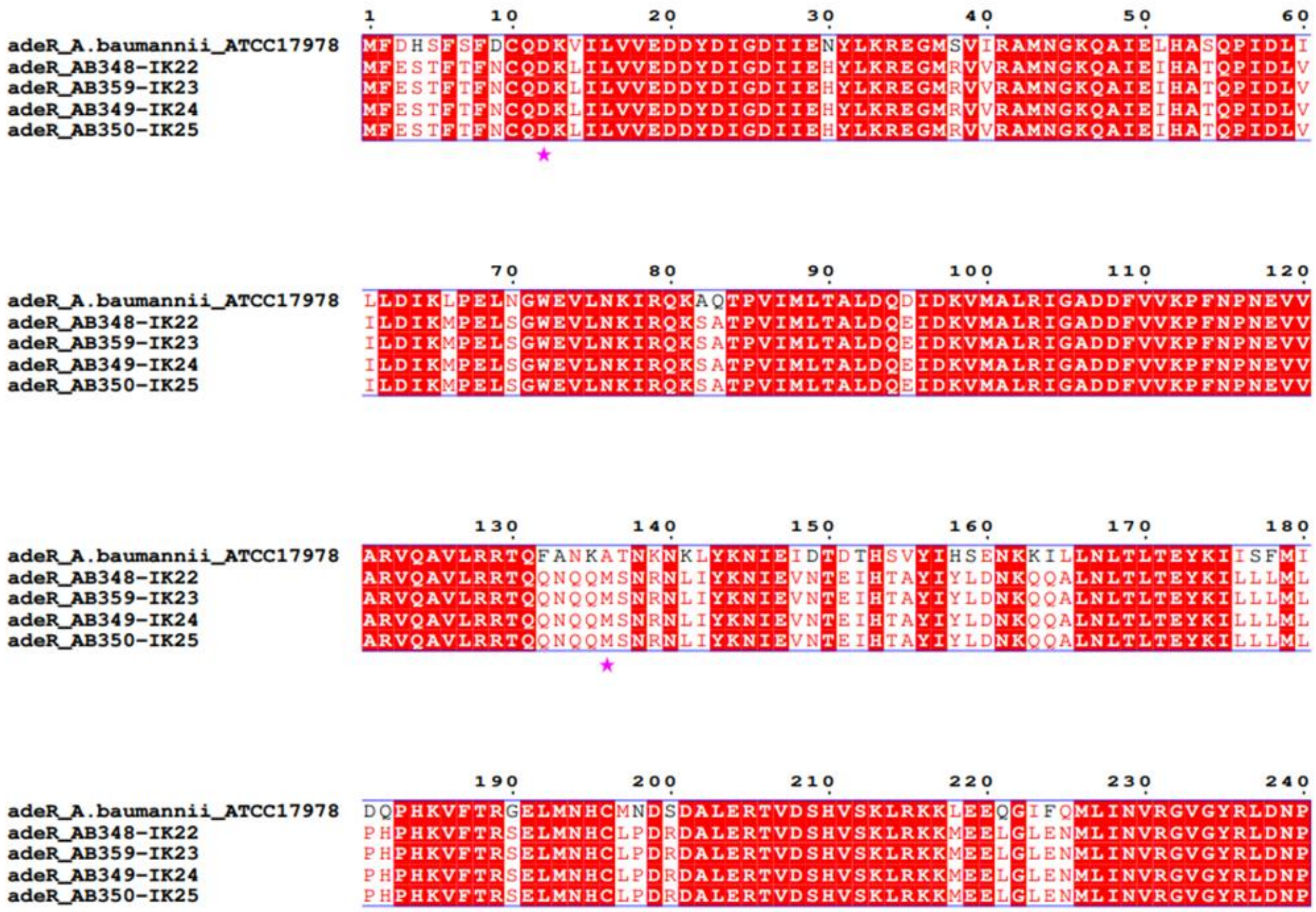

Fig. S11. Alignment of AdeR sequences from *A. baumannii* ATCC 17978-VU, *A. seifertii* DSM 102854^{T}, and the *A. seifertii* isolates (IK22, IK23, IK24, and IK25). The pink star indicates the location of a known mutation that confers colistin resistance in *A. baumannii* ATCC 17978-VU (D12I, A136V).

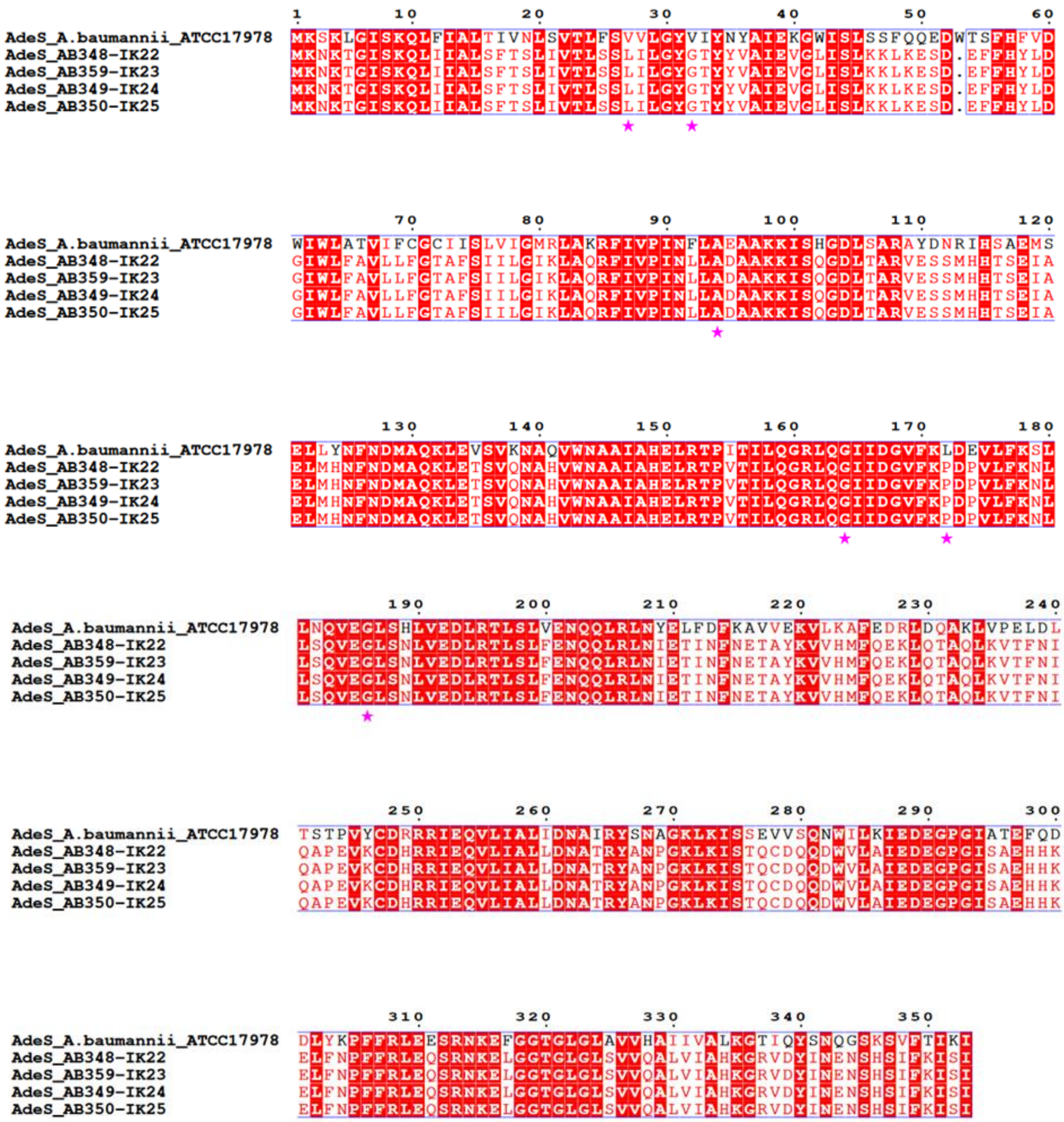

Fig. S12. Alignment of AdeS sequences from *A. baumannii* ATCC 17978-VU, *A. seifertii* DSM 102854^{T}, and the *A. seifertii* isolates (IK22, IK23, IK24, and IK25). The pink star indicates the location of a known mutation that confers colistin resistance in *A. baumannii* ATCC 17978-VU (V27I, V32L, A94V, G164A, L172P, G186V).

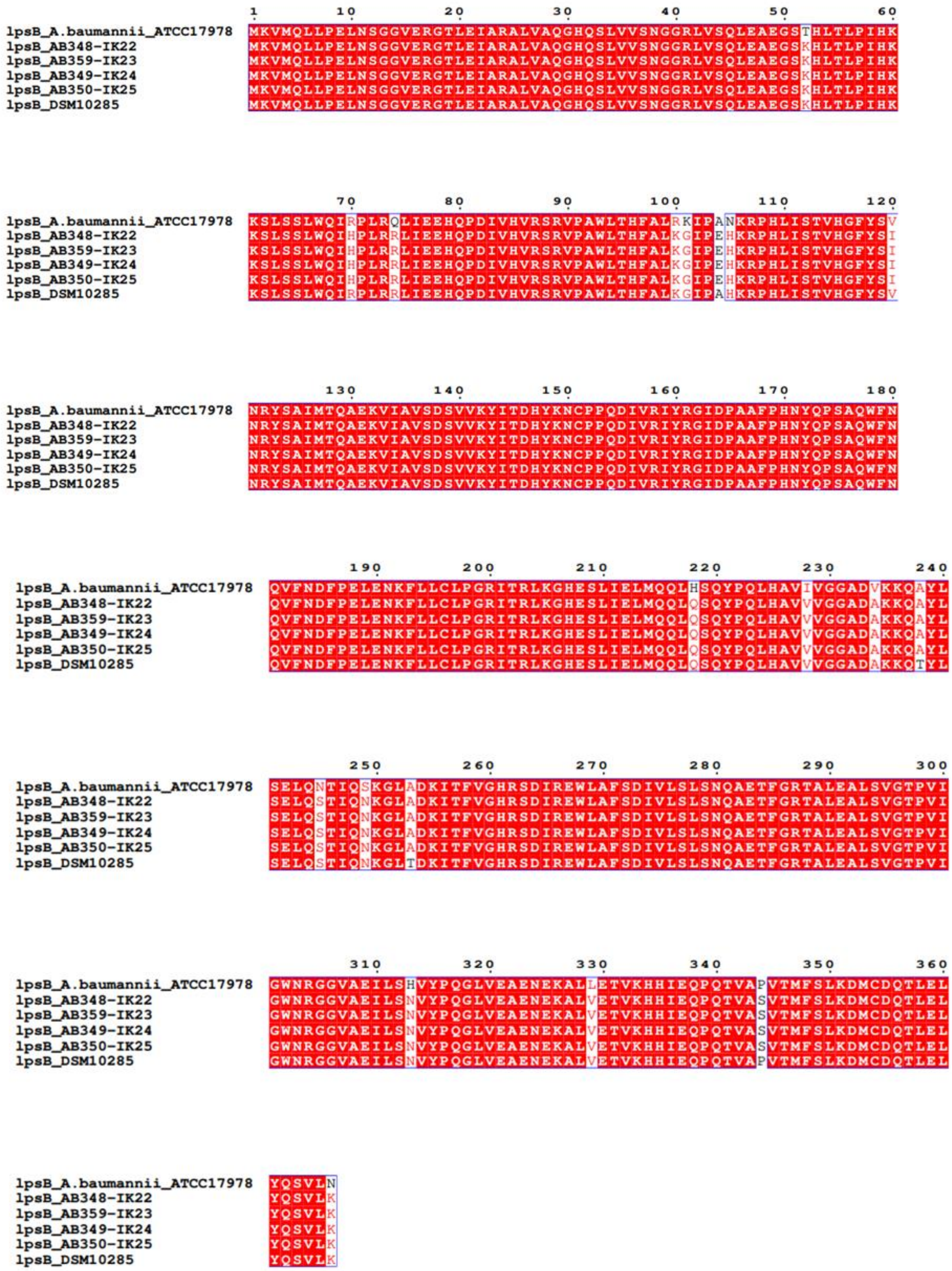

Fig. S13. Alignment of LpsB sequences from *A. baumannii* ATCC 17978-VU, *A. seifertii* DSM 102854^{T}, and the *A. seifertii* isolates (IK22, IK23, IK24, and IK25).

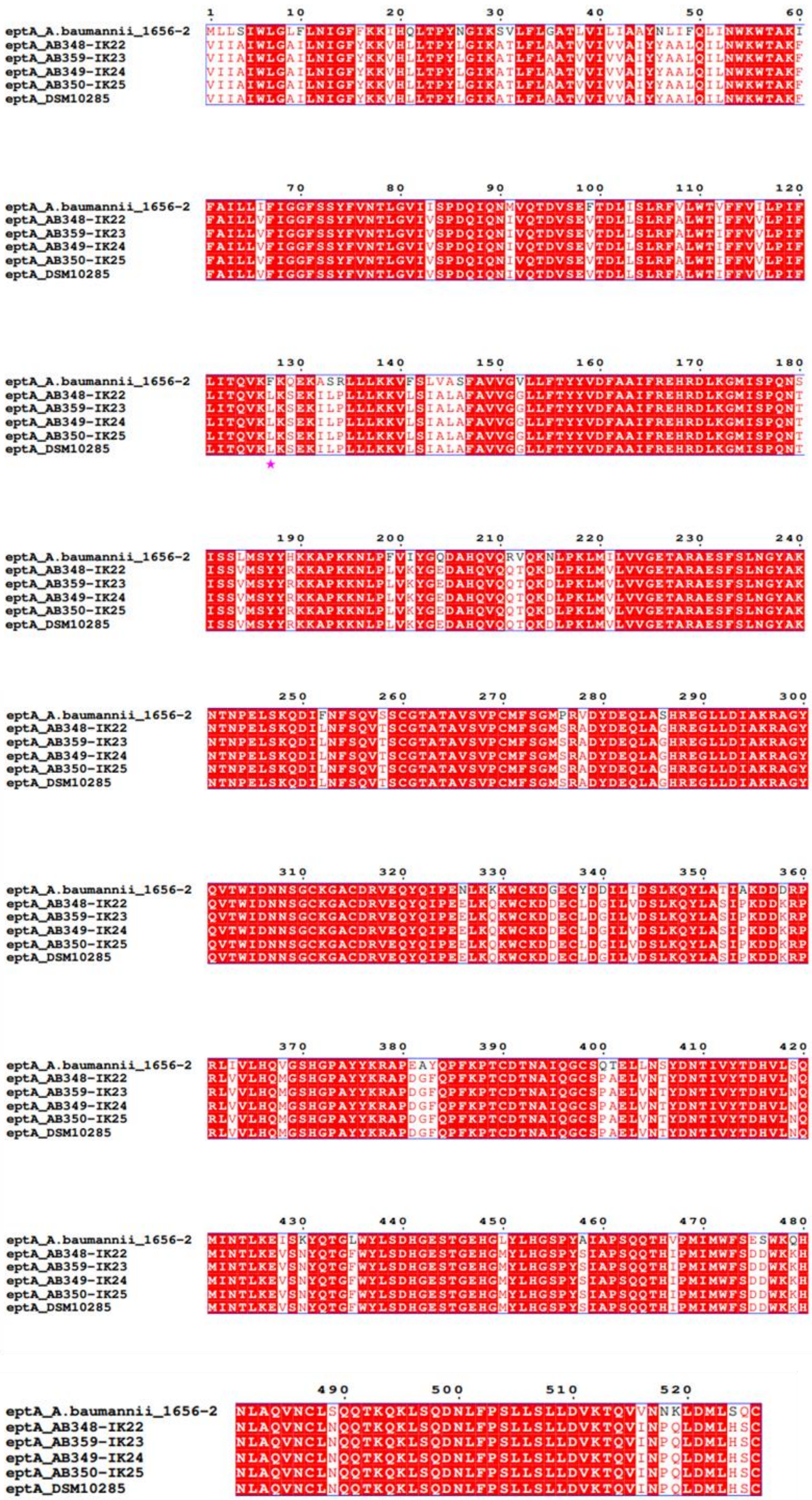

1 10 20 30 40 50 60
eptA_A.baumannii_1656-2 MLLSIWLGLFLNIGFFKKIHQLTPYNGIKSVLFLGATLVILIAAYNLIFQLINWKWTAKI
eptA_AB348-IK22 VIIAIWLGAILNIGFYKKVHLLTPYLGIKATLFLAATVVIVVAIYYAALQILNWKWTAKF
eptA_AB359-IK23 VIIAIWLGAILNIGFYKKVHLLTPYLGIKATLFLAATVVIVVAIYYAALQILNWKWTAKF
eptA_AB349-IK24 VIIAIWLGAILNIGFYKKVHLLTPYLGIKATLFLAATVVIVVAIYYAALQILNWKWTAKF
eptA_AB350-IK25 VIIAIWLGAILNIGFYKKVHLLTPYLGIKATLFLAATVVIVVAIYYAALQILNWKWTAKF
eptA_DSM10285 VIIAIWLGAILNIGFYKKVHLLTPYLGIKATLFLAATVVIVVAIYYAALQILNWKWTAKF
70 80 90 100 110 120
eptA_A.baumannii_1656-2 FAILLIFIGGFSSYFVNTLGVIISPDQIQNMVQTDVSEFTDLISLRFVLWTVFFVILPIF
eptA_AB348-IK22 FAILLVFIGGFSSYFVNTLGVIVSPDQIQNIVQTDVSEVTDLLSLRFALWTIFFVVLPIF
eptA_AB359-IK23 FAILLVFIGGFSSYFVNTLGVIVSPDQIQNIVQTDVSEVTDLLSLRFALWTIFFVVLPIF
eptA_AB349-IK24 FAILLVFIGGFSSYFVNTLGVIVSPDQIQNIVQTDVSEVTDLLSLRFALWTIFFVVLPIF
eptA_AB350-IK25 FAILLVFIGGFSSYFVNTLGVIVSPDQIQNIVQTDVSEVTDLLSLRFALWTIFFVVLPIF
eptA_DSM10285 FAILLVFIGGFSSYFVNTLGVIVSPDQIQNIVQTDVSEVTDLLSLRFALWTIFFVVLPIF
130 140 150 160 170 180
eptA_A.baumannii_1656-2 LITQVKFKQEKASRLLLKKVFSLVASFAVVGVLLFTYYVDFAAIFREHRDLKGMISPQNS
eptA_AB348-IK22 LITQVKLKSEKILPLLLKKVLSIALAFAVVGGLLFTYYVDFAAIFREHRDLKGMISPQNT
eptA_AB359-IK23 LITQVKLKSEKILPLLLKKVLSIALAFAVVGGLLFTYYVDFAAIFREHRDLKGMISPQNT
eptA_AB349-IK24 LITQVKLKSEKILPLLLKKVLSIALAFAVVGGLLFTYYVDFAAIFREHRDLKGMISPQNT
eptA_AB350-IK25 LITQVKLKSEKILPLLLKKVLSIALAFAVVGGLLFTYYVDFAAIFREHRDLKGMISPQNT
eptA_DSM10285 LITQVKLKSEKILPLLLKKVLSIALAFAVVGGLLFTYYVDFAAIFREHRDLKGMISPQNT
190 200 210 220 230 240
eptA_A.baumannii_1656-2 ISSLMSYYHKKAPKKNLPFVIYGQDAHQVQRVQKNLPKLMILVVGETARAESFSLNGYAK
eptA_AB348-IK22 ISSVMSYYRKKAPKKNLPLVKYGEDAHQVQQTQKDLPKLMVLVVGETARAESFSLNGYAK
eptA_AB359-IK23 ISSVMSYYRKKAPKKNLPLVKYGEDAHQVQQTQKDLPKLMVLVVGETARAESFSLNGYAK
eptA_AB349-IK24 ISSVMSYYRKKAPKKNLPLVKYGEDAHQVQQTQKDLPKLMVLVVGETARAESFSLNGYAK
eptA_AB350-IK25 ISSVMSYYRKKAPKKNLPLVKYGEDAHQVQQTQKDLPKLMVLVVGETARAESFSLNGYAK
eptA_DSM10285 ISSVMSYYRKKAPKKNLPLVKYGEDAHQVQQTQKDLPKLMVLVVGETARAESFSLNGYAK
250 260 270 280 290 300
eptA_A.baumannii_1656-2 NTNPELSKQDIFNFSQVSSCGTATAVSVPCMFSGMPRVDYDEQLASHREGLLDIAKRAGY
eptA_AB348-IK22 NTNPELSKQDILNFSQVTSCGTATAVSVPCMFSGMSRADYDEQLAGHREGLLDIAKRAGY
eptA_AB359-IK23 NTNPELSKQDILNFSQVTSCGTATAVSVPCMFSGMSRADYDEQLAGHREGLLDIAKRAGY
eptA_AB349-IK24 NTNPELSKQDILNFSQVTSCGTATAVSVPCMFSGMSRADYDEQLAGHREGLLDIAKRAGY
eptA_AB350-IK25 NTNPELSKQDILNFSQVTSCGTATAVSVPCMFSGMSRADYDEQLAGHREGLLDIAKRAGY
eptA_DSM10285 NTNPELSKQDILNFSQVTSCGTATAVSVPCMFSGMSRADYDEQLAGHREGLLDIAKRAGY
310 320 330 340 350 360
eptA_A.baumannii_1656-2 QVTWIDNNSGCKGACDRVEQYQIPENLKKKWCKDGECYDDILIDSLKQYLATIAKDDDRP
eptA_AB348-IK22 QVTWIDNNSGCKGACDRVEQYQIPEELKQKWCKDDECLDGILVDSLKQYLASIPKDDKRP
eptA_AB359-IK23 QVTWIDNNSGCKGACDRVEQYQIPEELKQKWCKDDECLDGILVDSLKQYLASIPKDDKRP
eptA_AB349-IK24 QVTWIDNNSGCKGACDRVEQYQIPEELKQKWCKDDECLDGILVDSLKQYLASIPKDDKRP
eptA_AB350-IK25 QVTWIDNNSGCKGACDRVEQYQIPEELKQKWCKDDECLDGILVDSLKQYLASIPKDDKRP
eptA_DSM10285 QVTWIDNNSGCKGACDRVEQYQIPEELKQKWCKDDECLDGILVDSLKQYLASIPKDDKRP
370 380 390 400 410 420
eptA_A.baumannii_1656-2 RLIVLHQVGSHGPAYYKRAPEAYQPFKPTCDTNAIQGCSQTELLNSYDNTIVYTDHVLSQ
eptA_AB348-IK22 RLVVLHQMGSHGPAYYKRAPDGFQPFKPTCDTNAIQGCSPAELVNTYDNTIVYTDHVLNQ
eptA_AB359-IK23 RLVVLHQMGSHGPAYYKRAPDGFQPFKPTCDTNAIQGCSPAELVNTYDNTIVYTDHVLNQ
eptA_AB349-IK24 RLVVLHQMGSHGPAYYKRAPDGFQPFKPTCDTNAIQGCSPAELVNTYDNTIVYTDHVLNQ
eptA_AB350-IK25 RLVVLHQMGSHGPAYYKRAPDGFQPFKPTCDTNAIQGCSPAELVNTYDNTIVYTDHVLNQ
eptA_DSM10285 RLVVLHQMGSHGPAYYKRAPDGFQPFKPTCDTNAIQGCSPAELVNTYDNTIVYTDHVLNQ
430 440 450 460 470 480
eptA_A.baumannii_1656-2 MINTLKEISKYQTGLWYLSDHGESTGEHGLYLHGSPYAIAPSQQTHVPMIMWFSESWKQH
eptA_AB348-IK22 MINTLKEVSNYQTGFWYLSDHGESTGEHGMYLHGSPYSIAPSQQTHIPMIMWFSDDWKKH
eptA_AB359-IK23 MINTLKEVSNYQTGFWYLSDHGESTGEHGMYLHGSPYSIAPSQQTHIPMIMWFSDDWKKH
eptA_AB349-IK24 MINTLKEVSNYQTGFWYLSDHGESTGEHGMYLHGSPYSIAPSQQTHIPMIMWFSDDWKKH
eptA_AB350-IK25 MINTLKEVSNYQTGFWYLSDHGESTGEHGMYLHGSPYSIAPSQQTHIPMIMWFSDDWKKH
eptA_DSM10285 MINTLKEVSNYQTGFWYLSDHGESTGEHGMYLHGSPYSIAPSQQTHIPMIMWFSDDWKKH
490 500 510 520
eptA_A.baumannii_1656-2 NLAQVNCLSQQTKQKLSQDNLFPSLLSLLDVKTQVVNNKLDMLSQC
eptA_AB348-IK22 NLAQVNCLNQQTKQKLSQDNLFPSLLSLLDVKTQVINPQLDMLHSC
eptA_AB359-IK23 NLAQVNCLNQQTKQKLSQDNLFPSLLSLLDVKTQVINPQLDMLHSC
eptA_AB349-IK24 NLAQVNCLNQQTKQKLSQDNLFPSLLSLLDVKTQVINPQLDMLHSC
eptA_AB350-IK25 NLAQVNCLNQQTKQKLSQDNLFPSLLSLLDVKTQVINPQLDMLHSC
eptA_DSM10285 NLAQVNCLNQQTKQKLSQDNLFPSLLSLLDVKTQVINPQLDMLHSC

Fig. S14. Alignment of EptA sequences from *A. baumannii* 1656-2*, *A. seifertii* DSM 102854^T, and the *A. seifertii* isolates (IK22, IK23, IK24, and IK25). The pink star indicates the location of a known mutation that confers colistin resistance in *A. baumannii* ATCC 17978-VU (F127L).

* *A. baumannii* ATCC 17978-VU has no gene annotated as *"eptA"*. *A. baumannii* 1656-2 was the top search result when the reference sequence from the Comprehensive Antibiotic Resistance Database was queried in BLASTn. The *eptA* sequence from this strain was used for tblastn and alignment.

LOS extraction and staining

The LOS extraction protocol was adapted from Marolda et al [6]. In brief, *A. seifertii* isolates and *A. baumannii* ATCC 17978-VU were grown to an OD_{600} of 1.5 and pelleted. Lysis buffer (2% w/v sodium dodecyl sulphate (SDS), 4% β-mercaptoethanol, 0.5 M of Tris-HCl (pH 6.8)) was added to each cell pellet and the samples were boiled. DNase I and proteinase K were added at a final concentration of 100 µg/mL and 1.2 mg/mL, respectively. After incubation with a hot phenol solution (90% phenol, 0.1% β-mercaptoethanol, 0.2% 8-hydroxyquinolone), the samples were chilled on ice and then centrifuged for phase separation. The aqueous phase was removed and washed with ethyl ether. The LOS extracts were then run on NuPAGE Bis-Tris gradient (4-12%) SDS-PAGE gels (Invitrogen, Waltham, USA) with NuPAGE MES running buffer (Invitrogen) and stained with the Pierce Silver Stain kit (Invitrogen) according to the manufacturer's protocol.

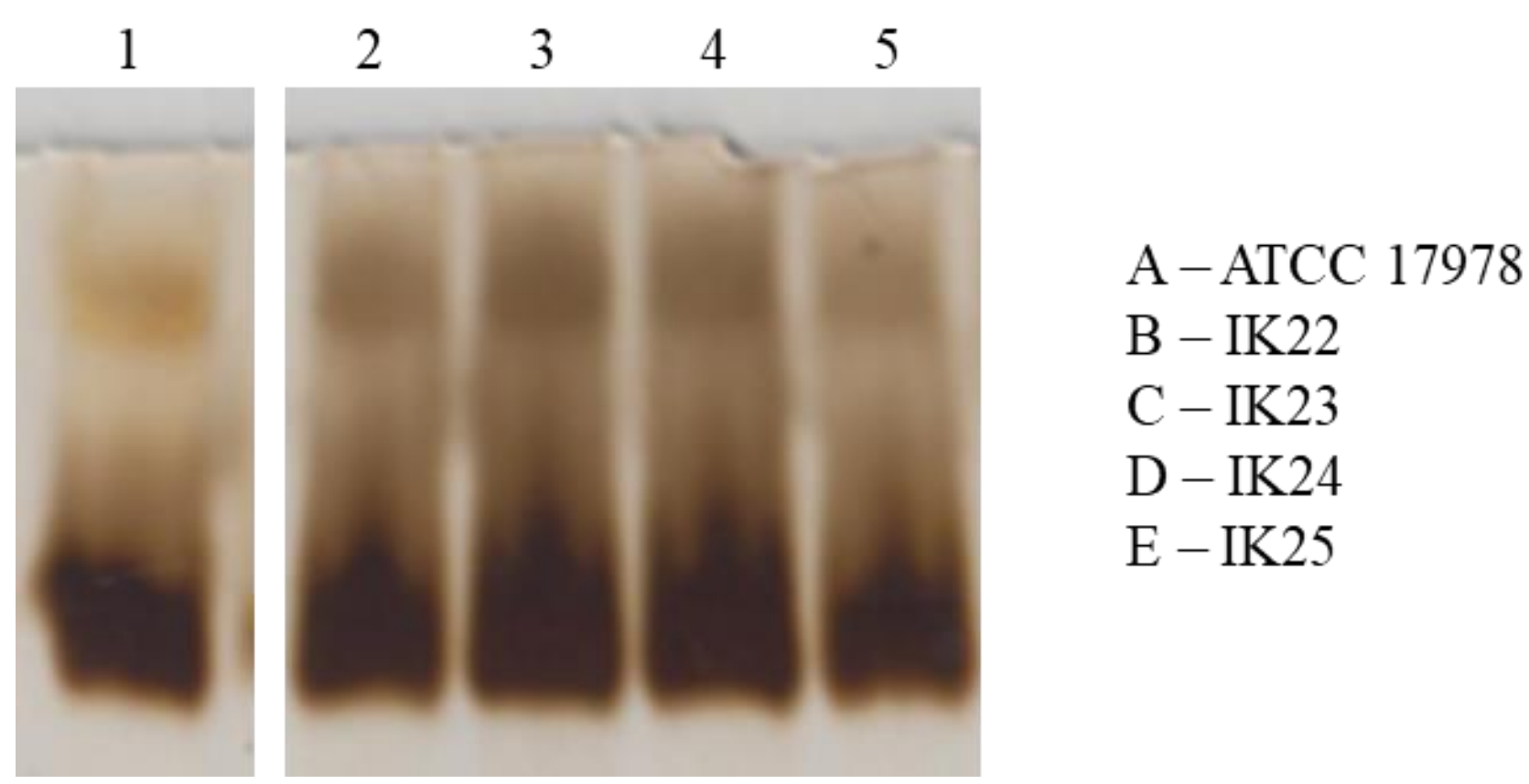

Fig. S15. **(d)** Silver-stained gel of extracted lipooligosaccharides from *A. baumannii* ATCC 17978-VU (1) and the *A. seifertii* isolates (2-5: IK22, IK23, IK24, and IK25, respectively).

Membrane permeabilization assay

Membrane integrity in the presence of colistin (0-16 µg/mL) was assessed using the fluorescent, non-polar dye 1-N-phenylnaphthylamine (NPN) according to Sabnis et al (2021) [7] and as detailed in the main manuscript.

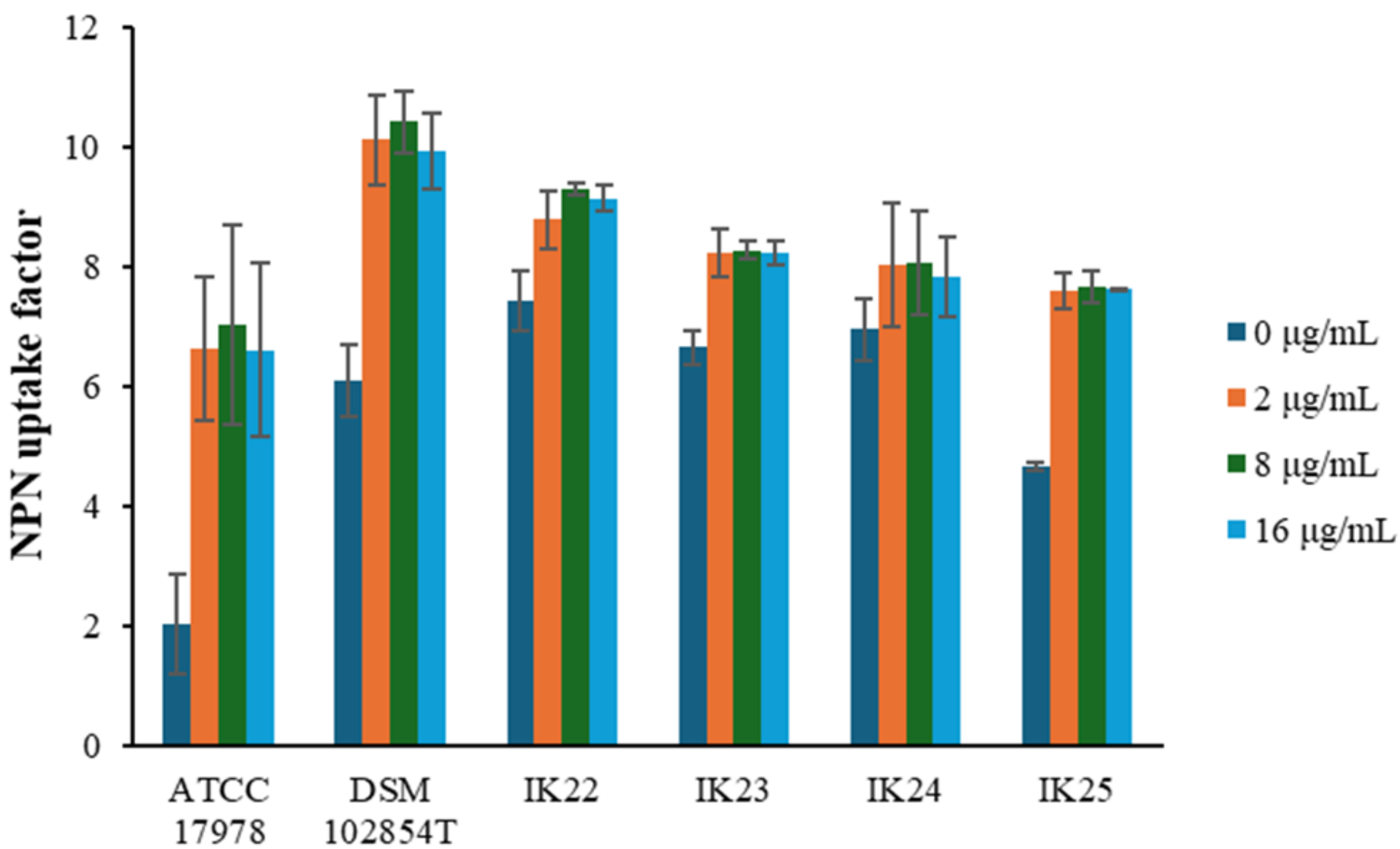

Fig. S16. Membrane integrity assessed for *A. seifertii* isolates (IK22, IK23, IK24, and IK25), type strain *A. seifertii* DSM 102854^{T}, and *A. baumannii* ATCC 17978-VU using 1-N-phenylnaphthylamine across an expanded range of colistin concentrations.

References

1. Sykes EME, Mateo-Estrada V, Engelberg R, Muzaleva A, Zhanel G, Dettman J, Chapados J, Gerdis S, Akineden Ö, Khan IUH, Castillo-Ramírez S, Kumar A. 2024. Phylogenomic and phenotypic analyses highlight the diversity of antibiotic resistance and virulence in both human and non-human *Acinetobacter baumannii*. mSphere 0:e00741-23.

2. Pfaffl MW. 2001. A new mathematical model for relative quantification in real-time RT-PCR. Nucleic Acids Res 29(9):e45.

3. Gertz EM, Yu Y-K, Agarwala R, Schäffer AA, Altschul SF. 2006. Composition-based statistics and translated nucleotide searches: improving the TBLASTN module of BLAST. BMC Biology 4:41.

4. Katoh K, Rozewicki J, Yamada KD. 2019. MAFFT online service: multiple sequence alignment, interactive sequence choice and visualization. Briefings Bioinf 20:1160-1166.

5. Robert X, Gouet P. 2014. Deciphering key features in protein structures with the new ENDscript server. Nucleic Acids Res 42:W320-W324.

6. Marolda CL, Lahiry P, Vinés E, Saldías S, Valvano MA. 2006. Micromethods for the characterization of lipid A-core and O-antigen lipopolysaccharide. Methods Mol Biol 347:237-252.

7. Sabnis A, Hagart KLH, Klockner A, Becce M, Evans LE, Furniss RCD, Al Mavridou D, Murphy R, Stevens MM, Davies JC, Larrouy-Maumus GJ, Clarke T, Edwards AM. 2021.Colistin kills bacteria by targeting lipopolysaccharide in the cytoplasmic membrane. eLife 10:e65836.
